# 2D Organic Materials: Status and Challenges

**DOI:** 10.1002/advs.202203889

**Published:** 2023-01-22

**Authors:** Xiaobing Yan, Ying Zhao, Gang Cao, Xiaoyu Li, Chao Gao, Luan Liu, Shakeel Ahmed, Faizah Altaf, Hui Tan, Xiaopeng Ma, Zhongjian Xie, Han Zhang

**Affiliations:** ^1^ School of Life Sciences, Institute of Life Science and Green Development, Key Laboratory of Brain‐Like Neuromorphic Devices and Systems of Hebei Province College of Electronic and Information Engineering Hebei University Baoding 071002 China; ^2^ Collaborative Innovation Center for Optoelectronic Science and Technology International Collaborative Laboratory of 2D Materials for Optoelectronics Science and Technology of Ministry of Education Institute of Microscale Optoelectronics College of Physics and Optoelectronic Engineering Shenzhen University Shenzhen 518060 P. R. China; ^3^ Department of Chemistry Women University Bagh Azad Kashmir Bagh Azad Kashmir Bagh 12500 Pakistan; ^4^ School of Materials Science and Engineering Georgia Institute of Technology North Avenue Atlanta GA 30332 USA; ^5^ Department of Respiratory Shenzhen Children's Hospital Shenzhen 518036 P. R. China; ^6^ Institute of Pediatrics Shenzhen Children's Hospital Shenzhen Guangdong 518038 P. R. China; ^7^ Shenzhen International Institute for Biomedical Research Shenzhen Guangdong 518116 China

**Keywords:** 2D organic materials, application, preparation methods, properties, synthesis

## Abstract

In the past few decades, 2D layer materials have gradually become a central focus in materials science owing to their uniquely layered structural qualities and good optoelectronic properties. However, in the development of 2D materials, several disadvantages, such as limited types of materials and the inability to synthesize large‐scale materials, severely confine their application. Therefore, further exploration of new materials and preparation methods is necessary to meet technological developmental needs. Organic molecular materials have the advantage of being customizable. Therefore, if organic molecular and 2D materials are combined, the resulting 2D organic materials would have excellent optical and electrical properties. In addition, through this combination, the free design and large‐scale synthesis of 2D materials can be realized in principle. Furthermore, 2D organic materials exhibit excellent properties and unique functionalities along with great potential for developing sensors, biomedicine, and electronics. In this review, 2D organic materials are divided into five categories. The preparation methods and material properties of each class of materials are also described in detail. Notably, to comprehensively understand each material's advantages, the latest research applications for each material are presented in detail and summarized. Finally, the future development and application prospects of 2D organic materials are briefly discussed.

## Graphical abstract

Organic molecular and two dimensional (2D) materials combined will result into 2D organic materials, which would have unique optical and electrical properties. The free design and large scale synthesis of 2D materials can be realized in principle. This work summarizes status and challenges of 2D organic materials, and the great potential for developing sensors, biomedicine, and electronics.

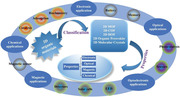

## 1 Introduction

The advent of graphene has sparked great interest in the study of other innovative 2D materials, which have become a hot research topic.^[^ ^1^, ^2^, ^3^, ^4^ ^]^ The novel inherent physical characteristics of graphene (electronic, mechanical and heat transfer characteristics) make it a pioneer 2D material for the development of science and technology and other practical applications.^[^ ^5^, ^6^ ^]^ Interestingly, the research on 2D materials is still in the development stage, so the number of 2D material libraries is growing year by year with new materials being introduced every year. Currently, such libraries comprise more than 150 materials.^[^ ^7^ ^]^ In addition to the excellent characteristics of graphene, the new 2D materials exhibit extraordinary potential in biomedicine, sensors, transistors, light‐emitting diodes (LEDs) and catalysis.^[^ ^8^, ^9^, ^10^, ^11^, ^12^ ^]^ The emergence of these new 2D materials can solve the application limitations of different devices. Even though 2D materials have been previously researched because of their atomic‐scale thickness and unique photoelectric properties, some challenges remain to be solved: i) continuous development of new 2D materials and their structures, which could subsequently induce prominent or significant physical, chemical, mechanical, and optoelectronic properties, and ii) the use of different processing methods to prepare large‐area and low‐cost 2D materials.^[^ ^13^, ^14^ ^]^ The term preparation is often used to express the action or process of making ready to get almost a known compound by a known method. The term synthesis often expresses combination or composition, in particular, the installation or creation of a new compound by a new method. 2D organic materials show unique advantages in meeting the above‐mentioned challenges. For example, organic molecules have tailorable properties that can realize the free design of 2D materials and substantially enrich the number of 2D material families, thus solving the first challenge.^[^ ^15^ ^]^ The small organic molecules are connected by noncovalent bonds and have excellent self‐assembly capabilities; thus, large‐area, high‐quality 2D crystals can be prepared via solution processing methods, solving the second challenge.^[^ ^15^ ^]^ Moreover, organic materials have many advantages, such as intrinsic flexibility, amenability of 2D materials toward top‐down and bottom‐up lithography methods; their pliability and ability to be mechanically strained to create new structure–property–function relationships; and their unique chemistry, with large surface areas and low weight.^[^ ^16^, ^17^ ^]^ Therefore, 2D organic materials have obvious advantages.

**Figure 1:**
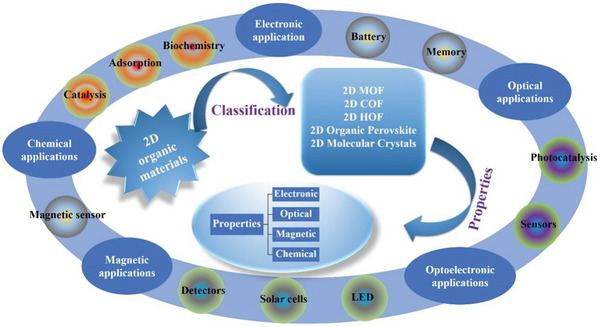
Classification of 2D organic materials and related applications.

In existing reviews, the research findings are based on a single type of 2D material or certain characteristics, such as the study of 2D transition metal sulfides,^[^ ^18^ ^]^ atomic defects in 2D materials^[^ ^19^ ^]^ and 2D hybrid perovskite.^[^ ^20^ ^]^ These are detailed studies within specific fields, but their research content is limited, and no comparison and summary exist from a broad scope. In this review, considering the entire subject of 2D organic material as a starting point, it is divided into metal‐organic frameworks (MOFs), covalent‐organic frameworks (COFs), hydrogen‐bonded organic frameworks (HOFs), perovskite and molecular crystals according to different structural properties (**Figure** **1**). The synthesis, preparation methods, properties and applications in electronics, sensors, biomedicine and other fields of all five types of 2D organic materials, as classified in this study, are explored in detail. Additionally, the challenges and prospects of 2D organic materials are briefly analyzed.

## 2 2D Organic Materials

2D organic materials are considered the most attractive new class materials. They have always been the focus of research by scientists and have been widely used in various fields.^[^ ^12^, ^21^ ^]^ Herein, 2D organic materials are classified into five classes. This section briefly describes each class. The term preparation is often used to express the action or process of making ready to get almost a known compound by a known method.

### 2.1 Metal‐Organic Frameworks (MOFs)

Many researchers are very interested in studying 2D MOFs. MOFs are porous crystalline materials, formed by self‐assembled metal ions and organic ligands. The continuous research and studies on them reveal that there are > 2 × 10^4^ MOFs, where different metal ion‐center and organic junction(s) are linked. MOFs can be grown into different 3D, 2D, 1D, and 0D forms. They can also be used as active materials for electrocatalysis and energy storage applications. Notably, MOFs can be transformed into a huge spectrum of functional materials through various methods, such as pyrolysis and chemical and physical treatments.^[^ ^22^ ^]^

2D MOFs being a branch of MOFs bear many important characteristics of this family. They combine the advantages of MOFs and 2D structure, and show more prominent characteristics in catalysis, bionic enzyme and sensor applications. However, the synthesis processes of 2D MOFs are relatively difficult and time‐consuming.^[^ ^23^ ^]^ The most common members of the 2D MOF family are isoreticular MOFs (IRMOFs),^[^ ^24^ ^]^ zeolite imidazolate frameworks (ZIFs)^[^ ^25^, ^26^, ^27^, ^28^ ^]^ and materials of institute Lavoisier (MIL).^[^ ^29^ ^]^ ZIF is often used as a solid‐phase precursor of carbon nanomaterials, and MIL is widely accepted by scholars because of its large specific surface area and stable structure.

### 2.2 Covalent‐Organic Frameworks (COFs)

2D COFs are porous materials that have attracted increasing research attention because of their interesting structure, like an organic framework, adjustable porosity and predictable framework. When compared with short‐range covalent polymers connected by irreversible condensation, COFs differ considerably.^[^ ^30^ ^]^ 2D COFs reflect a very ordered crystal structure formed through a reversible reaction.^[^ ^31^ ^]^ Generally, conventional crystalline porous solids (such as zeolite^[^ ^32^, ^33^ ^]^) have precise predesigned structures, and can achieve function‐oriented structure and chemical control.^[^ ^34^ ^]^ Additionally, 2D COFs have many advantages, such as structural multiplicity,^[^ ^35^ ^]^ low density,^[^ ^36^ ^]^ high thermal stability^[^ ^37^, ^38^ ^]^ and permanent porosity.^[^ ^39^ ^]^ Herein, three aspects of 2D COFs are elaborated by considering their unique structure.

The first category includes highly porous 2D crystalline materials comprising light elements (C, H, N, O, and B) with large surface areas, such as B‐COF, COF‐1, COF‐5, COF‐102 and COF‐103.^[^ ^40^, ^41^, ^42^, ^43^, ^44^, ^45^, ^46^, ^47^ ^]^ These special properties enable 2D COFs to be used for gas separation and storage applications.^[^ ^40^, ^41^, ^42^, ^43^ ^]^ Crystalline 2D COFs comprising 2D COF‐102, COF‐103, COF‐105 and COF‐108 structures are a product of co‐condensation and self‐condensation formed by a rigid molecular structure. These structures comprise four sides (2a–c).

**Figure 2:**
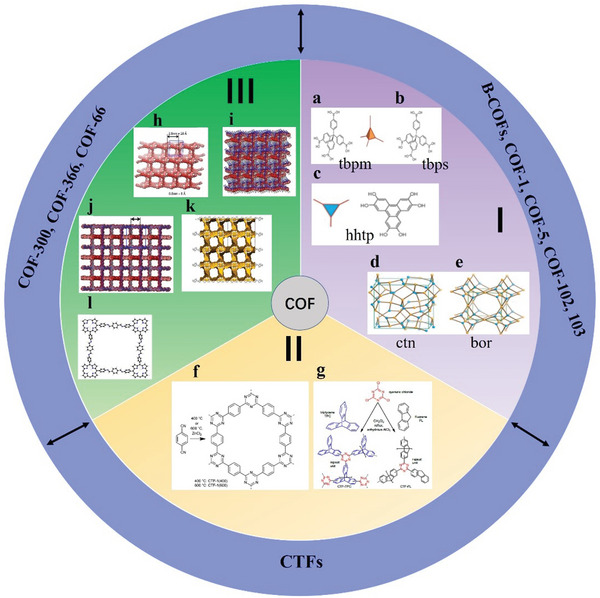
a,b) Tetrahedral and c) triangular structures (orange polyhedron and blue triangle, respectively). These structures are shown online in c) ctn and d) bor. Reproduced with permission.^[^ ^45^ ^]^ Copyright 2007, American Association for the Advancement of Science. f,g) Structure of CTF‐1, CTF‐TPC and CTF‐FL. Reproduced with permission.^[^ ^55^, ^56^ ^]^ Copyright 2015, The Royal Society of Chemistry. h–k) Rhomboid symmetry, interpenetrating structure and pore diameter, respectively. Reproduced with permission.^[^ ^57^ ^]^ Copyright 2016, Wiley‐VCH. l) Unit cell of COF‐366. Reproduced with permission.^[^ ^58^ ^]^ Copyright 2013, Elsevier Ltd.

The second category includes the covalent triazine framework (CTF). CTFs have the advantages of a large surface area ratio and high thermal stability.^[^ ^48^, ^49^ ^]^ CTFs are cheap and easy‐to‐obtain original materials that are easily synthesized and have a certain hydrophilicity. **Figure** **2**f,g shows the structure of CTF‐1, CTF‐TPC and CTF‐FL.

The third category includes 2D COF‐300, COF‐3, and COF‐66. COF‐300 are crystalline and microporous COFs connected by imines. They have large surface areas of ≈1400–1500 m^2^ g^−1^ and are highly crystalline.^[^ ^44^ ^]^ The pore diameter of the hole is ≈8 Å for interpenetrating structures and 28–30 Å for noninterpenetrating structures (Figure 2h–k). Herein, these structures are classified as amine‐based 2D COFs mainly because imine functional groups bind them together. According to the inspiration of the internal zeolite reaction, this material could be used as the main component of catalysts, such as those used in cross‐coupling reactions.^[^ ^50^, ^51^, ^52^, ^53^ ^]^ The relevant parameters of the unit cell of 2D COF‐366 are *a* = *b* = 25.696 Å and *c* = 12.541 Å with the P4/m space group. Its unit cell comprises 16 N, 120 C and 76 H atoms.^[^ ^54^ ^]^ The AA stacking sequence is formed by the 2D COF‐366 crystal structure. The AA distance between each layer is 6.27 Å, and the hole diameter is 20 Å.^[^ ^54^ ^]^ Tetra(p‐aminophenyl) porphyrin (TAPP) with a periodic framework contains a bond with terephthalaldehyde in each layer (Figure 2l).

### 2.3 Hydrogen‐Bonded Organic Frameworks (HOFs)

Intermolecular hydrogen bonds are the bridge of organic molecules; simultaneously, crystalline materials assembled under the synergistic action of *π*–*π* stacking and other intermolecular forces are called HOFs.^[^ ^59^, ^60^, ^61^, ^62^, ^63^ ^]^ In 1997, Wuest et al. constructed a crystalline network structure connected by hydrogen bonds through small organic molecules containing 2,4‐diaminotriazinyl (DAT) motifs.^[^ ^64^ ^]^ Until 2005, Sozzani et al. constructed the first 2D HOF materials with stable structures and permanent holes, thus actualizing the development of HOF materials.^[^ ^65^ ^]^ As an important part of HOF materials, 2D hydrogen‐bonded organic materials have unique superiorities of mild synthesis conditions, high crystallinity, strong solvent machinability and large specific surface area; additionally, they have potential applications in many fields.^[^ ^60^, ^66^, ^67^, ^68^, ^69^ ^]^ After several years of development, various small organic molecules with hydrogen bond donors or acceptors have been developed to construct 2D HOF materials. However, the skeleton often collapses after losing solvent molecules owing to the low bond energy of hydrogen bonds; hence, the synthesis of 2D HOFs materials with permanent pores is a problem that requires resolution.^[^ ^70^ ^]^ Recently, the synthesis of 2D HOF materials with a new structure, new functions, unique properties and lasting porosity has been one of the main topics of research in porous materials.

### 2.4 Perovskite

Perovskite is recognized as the most promising new‐generation photovoltaic material; its structural formula is ABX_3_. The perovskite family can be expanded by replacing A, B and X with different materials.^[^ ^71^ ^]^ Of these, organic perovskite materials are formed by replacing A with organic cations, and 2D organic perovskite materials are obtained using different methods.^[^ ^72^ ^]^ Currently, scientists have studied more 2D organic perovskite materials as follows. First, according to different monovalent cations A, it can be divided into (MA)BX_3_ (MA^+^ = CH_3_NH_3_^+^), (PEA)BX_3_ (PEA is phenyl ethyl ammonium) and (FA)BX_3_ (FA^+^ = HC(NH_2_)_2_^+^). Then, according to different divalent cations B (mainly Pb^2+^and Sn^2+^), it can be divided into APbX_3_ and ASnX_3_. Finally, according to different anions X (mainly halide ions), it can be divided into ABI_3_, ABCl_3_ and ABBr_3_.^[^ ^72^ ^]^ They roughly include all 2D organic perovskite materials.

The 2D perovskites materials are much more stable as compared to 3D due to organic ligands protection. Also as compared to 3D, 2D perovskites possess natural quantum well. Wider energy band and good ambient stability. 2D perovskites possess several tremendous properties such as control quantum wells width. Ultrafast transfer of energy and dense film formation, which shows excellent potential in the field of optoelectronics and spintronics. The combination of organic–inorganic hybrid lead halide perovskite materials would bring tremendous progress in light‐emitting and photovoltaics applications. The efficiency of perovskite solar cells is above 25%, whose power conversion quite better than best silicon solar cell. Additionally, asymmetric lattice structures and extra spacing cations give extra degree of freedom, which enhanced intrinsic physical properties such as exciton binding energy, optical bandgap, and dielectric constant. The photophysical behavior such as charge carrier transport, exciton dynamics and electron–phonon coupling is sturdily enhanced the performance of LEDs and solar cells. The 2D perovskites have strong optical anisotropy, where dipole moments polarized in inorganic framework. Also 2D perovskite crystals at grain boundaries have edge states, which caused exciton dissociation into the free carriers. Like 3D perovskites, 2D perovskites also possess strong electron–phonon coupling. This belongs to ionic nature and materials stiffness.^[^ ^73^, ^74^ ^]^

Currently, most research on 2D organic perovskite materials is performed in the field of optoelectronics, especially for solar cells. Related reports continuously explore the photoelectric conversion efficiency and search for materials with low cost, high efficiency and good film‐forming ability. Additionally, because 2D organic perovskite materials have excellent electrical and optical properties,^[^ ^75^ ^]^ a new wave of research has been launched in many other fields, such as electronic devices and photovoltaics.^[^ ^76^, ^77^ ^]^

### 2.5 Molecular Crystals

Molecular crystals are a new type of 2D organic electronic material with highly uniform morphology, horizontally continuous atoms and highly ordered molecules. They are one of the most popular material candidates in recent years. Molecular crystals have an interface interaction that can be well confined in a 2D range, showing the effects of different layers and increasing the efficiency and quality of carrier injection and modulation. Moreover, they have the advantages of minimum charge trap concentration, perfect structure and no grain boundaries. Additionally, 2D molecular crystals effectively enhance field‐effect transistor performance owing to their ultra‐thin structural characteristics and excellent interface quality. 2D molecular crystals have attractive application prospects in advanced electronic technology because of their unique advantages, lightweight structure, material versatility and chemical and environmental stability. Molecular crystals provide new insight into the generation of layered and heterostructures.

### 2.6 The Solution‐Processed COFs and Amorphous Porous Materials

**Figure 3:**
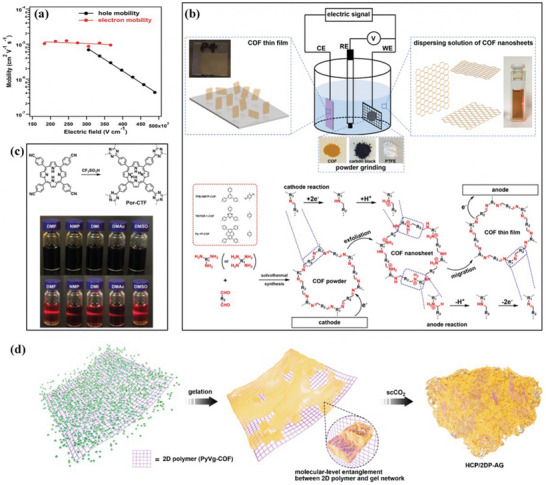
a) Dependence of the hole (black line) and electron (red line) mobility in the COF films on the electric field in the vertical direction by a TOF method. Reproduced with permission.^[^ ^78^ ^]^ Copyright 2019, Royal Society of Chemistry. b) Electrocleavage synthesis of solution‐processed, imine‐linked, and crystalline covalent organic framework thin films. Reproduced with permission.^[^ ^79^ ^]^ Copyright 2022, American Chemical Society. c) Schematic representation of protonated TPCP. Reproduced with permission.^[^ ^80^ ^]^ Copyright 2021, Wiley‐VCH. d) Photographs of the TPCP dissolved in various solvents. Upper: concentrated solutions under sunlight; lower: dilute solutions under 365 nm UV light showing the Tyndall effect. Reproduced with permission.^[^ ^82^ ^]^ Copyright 2022, American Chemical Society.

In order to design the next generation of 2D materials, covalent organic frameworks (COFs) are emerging materials. COFs are used as processable and insoluble solids for thin film preparation in optoelectronics applications. The highly soluble crystalline COF material is prepared by interlayer interactions. These COFs are significantly soluble in several organic solvents and form truly stable solution form. The solution process‐ability COFs with unique features of large area, high quality can be done on several substrates in efficient manner with control thickness. These materials with such compatibility are used in various device applications. COFs films show electrically anisotropic behavior, which means its intralayer has rare carrier conduction whereas the interlayer has the highest conduction rate, as shown in **Figure** **3**a which opens a novel path for the high‐performance COFs with diverse functions for optoelectronic devices such as transistors, diodes, photocatalysis and energy storage.^[^ ^78^ ^]^ Initially, synthesis of covalent organic frameworks (COFs) thin films was a major challenge. For this purpose, an electrocleavage synthesis strategy was introduced, in which at room temperature from electrolyte solutions COF films is directly linked on electrodes, as shown in Figure 3b. This caused COFs cathodic exfoliation, which converts COF powders into nanosheets through electrochemical reduction and protonation. In which nanosheets move towards anode producing COFs structures by anodic oxidation. Such kinds of COF s films provide excellent platform to promoting mass transfer and also have high rate constants due to extraordinarily rapid iodine adsorption.^[^ ^79^ ^]^ Another method is to make TPCP solutions, in which synthesis of extremely soluble proton‐exfoliated TPCP into the TPCP skeletons is done. This TPCP solution contains excellent thermoelectric properties. Here charged colloidal spheres possess electrostatic repulsive behavior. Such TPCP charges make ionic bonds, which are highly soluble and cause processability into high‐quality films highest power factor. These significant results an excellent platform for thermoelectric materials.^[^ ^80^ ^]^ Generally, proton‐conducting membranes in 3D form are used in fuel cells. Now a day's a new strategy is being used by combing material flexibility and solution processing into porous and amorphous polymers. We prepare nanoporous polymer as the proton‐accepting site to generate heavy charges upon polymer skeletons, which makes organic polymers to significantly disperse into the organic solvents and make amorphous and uniform membranes. Such kinds of membranes contain proton conductivity of 0.30 S cm^−1^ (298 K and 90% relative humidity), low resistance of 3.02 Ω, and a H^+^ transport number of 0.98 that was very close to the upper limitation of 1.0.^[^ ^81^ ^]^ Hyper cross‐linked polymers (HCPs), in 3D shape, have covalent organic linkage and are promising materials due to easy functionalization and large surface area. But such materials have low processability due to linkage rigidity on covalent crosslinking. But still solution processability for several applications is still challenging. Generally, HCPs form insoluble powder rather than gels. The HCP gels from a thermally induced polymerization, which makes a solubilization, covalent bond. This gel contains hierarchical porosities and mechanical stiffness. Such HCP gels are also used for molecular‐level hybridization with 2D polymers during the formation of HCP gel. This procedure forms functional gels and aerogels, which increase the mechanical stiffness and porosities, as shown in Figure 3d. Hybrid gels can also be used for water contaminants separators with the efficiency of 97.9 and 98.6% for methylene blue and KMnO4, respectively. These results revealed the HCP gel potentials and their hybrid derivatives are used in separation systems requiring macroscopic scaffolds with hierarchical porosity.^[^ ^82^ ^]^

### 2.7 Others

2D organic materials have always been the focus of scientific research. In addition to the above five categories, some materials belong to 2D organic polymers, such as 2D hyper‐cross‐linked polymer (HCP), 2D conjugated microporous polymer (CMP) and 2D inherently microporous polymer (PIM).^[^ ^83^ ^]^ Porous polymers can be designed and prepared at the molecular level, and have a controllable surface area along with a good pore structure, which is conducive to combining various chemical functional groups into a porous structure. Because of the diversity of the structure of 2D organic materials, they may be used to optimize electronic conductivity and higher carrier conductivity, for example, 2D self‐assembled monolayers (SAMs).^[^ ^84^ ^]^ SAMs can form a monolayer molecular film through the self‐assembly of surfactant molecules, and the head and tail groups of the constituent molecules can be tailored to obtain desired properties.

## 3 Synthesis of 2D Organic Materials

In existing research reports, most 2D organic materials are obtained from the bulk counterpart.^[^ ^85^ ^]^ This synthetic method considerably limits the discovery of 2D organic materials and the practical application of 2D material in various fields also in addition to posing huge challenges.^[^ ^86^ ^]^ Therefore, scientists are continuously exploring better preparation methods for 2D organic materials, such as the solution, solvent evaporation and diffusion methods. The term synthesis often expresses combination or composition, in particular, the installation or creation of a new compound by a new method. This review explains the synthesis methods of 2D organic materials in detail and discusses more options and challenges for the future of this emerging field.

### 3.1 Synthesis of MOF

The synthetic methods of 2D MOFs are mainly distributed into two types, top‐down and bottom‐up approaches, which will be introduced in this section. Top‐down methods include ultrasonic stripping and interface synthesis. Bottom‐up methods include the surfactant‐assisted, soft template‐assisted and template methods.^[^ ^87^ ^]^

The mechanism behind the use of 2D materials is that they possess exceptional optical, thermal, mechanical, and electrical properties. These materials include thin sheets, monomer units, covalent in‐plane bonding and layer‐substrate bonding leads to distinguished chemical and physical properties, such as catalysis, sensing, separation, energy storage and conversion, and other related fields.

Also, 2D nanostructures of organic materials possess distinctive features in biofunctionality and are quite sensitive to bioanalytes, Which help to design highly effective biosensors. The 2D organic structure with peculiar properties, such as lightweight, easy production, low cost, and ecofriendly nature, extend their scope in several 2D nanodevices. The organic materials are outstandingly suited to produce sustainable, bendable, and biodegradable ultrathin electronics. Good accessibility of bio/chemical‐functionalities and material softness, 2D organics promise high potential for use in bio/chemical sensing applications. However, like to inorganic 2D materials, 2D organics materials and nanodevices are not much‐studied yet.^[^ ^88^, ^89^ ^]^

#### 3.1.1 Top–Down Synthesis of 2D MOFs

**Figure 4:**
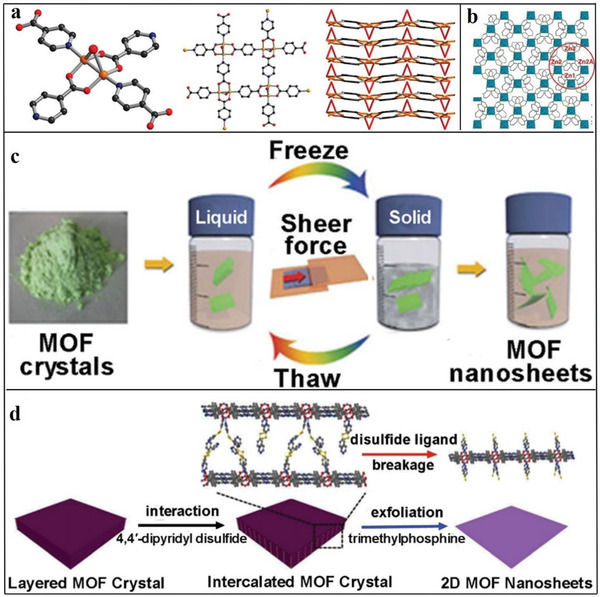
a) Position of Cu in [Cu_2_Br(IN)_2_]*_n_*, construction of monolayer [Cu_2_Br(IN)_2_]*_n_* and heaping of the layer down the *ɑ*‐axis. Reproduced with permission.^[^ ^92^ ^]^ Copyright 2010, Royal Society of Chemistry. b) Monolayer Zn_2_(bim)_4_ nanometre sheet structural graph. Blue represents the irregular polyhedron of zinc coordination, and the bar shape represents the bim ligand. Reproduced with permission.^[^ ^93^ ^]^ Copyright 2014, American Association for the Advancement of Science. c) Schematic of freezing and thawing process of MAMS‐1 crystal spalling into dispersed nanosheets. Reproduced with permission.^[^ ^95^ ^]^ Copyright 2017, Nature Publishing Group. d) Definition graph of 2D MOF nanosheet synthesis by intercalation and chemical spalling. Reproduced with permission.^[^ ^94^ ^]^ Copyright 2017, American Chemical Society.

The top‐down method mainly resists the interlayer interactions of layered MOF and peels the layered MOF into 2D nanometer sheets.^[^ ^90^, ^91^ ^]^ For example, Zamora et al. prepared [Cu_2_Br(IN)_2_]*_n_* by a top‐down ultrasonic stripping method, which is 2D MOF.^[^ ^87^ ^]^ In the structure of the product demonstrated in **Figure** **4**a, the layered structure comprises a copper dimer, bromide ligands and four isonicotinic acid ligands. The 2D layer along the *ɑ* shaft is stacked to form the final crystalline state.^[^ ^92^ ^]^ Yang et al. stripped a monolayer of 2D MOFs (Zn_2_(bim)_4_) using a top‐down method.^[^ ^93^ ^]^ The original large MOF crystals are stacked along the axis by interlamellar van der Waals forces. As shown in Figure 4b, a layer of Zn_2_(bim)_4_ comprises an irregular geometry formed by zinc ions and four benzimidazole ligands. Large MOF crystals are formed by stacking the layers through Van der Waals forces. First, the original big‐sized crystal was ground by a rotating wet ball at 60 rpm, and stripped using ultrasonic technology. During the stripping process, Yang et al. used a compound of methyl alcohol and n‐propanol (volume rate = 1:1). The grinding process accelerated the spread of methanol molecules into the crystal, and the n‐propanol adhered to the surface of the detached nanometer sheet, thereby protecting the nanometer sheet. Zhao et al. used a top‐down method to synthesize Ni_8_(5‐BBDC)_6_(µ‐OH)_4_ (beseeched as MAMS‐1).^[^ ^99^ ^]^ The main method entails adding MAMS‐1 crystal powder to n‐hexane, freezing the solution in liquid nitrogen at ‐196 °C and putting it in water at 80 °C. During the conversion of n‐hexane in the solid and liquid states, a cutting force can be generated to act on the MAMS‐1 crystal, transforming it into a nanosheet structure (Figure 4c). Zhou et al. also used a top‐down approach to strip out MOF nanocrystals.^[^ ^94^ ^]^ First, layered Zn_2_(PdTCPP) was produced, and dipyridine ligands (4,4ʹ‐dipyridine disulfide (DPDS)) were inserted as intercalators into the MOF to obtain intercalated crystal Zn_2_(PdTCPP)(DPDS). The coordination between DPDS and metal crystals weakens the interlayer interaction of the MOF. Then, the disulfide bond is detrimethyl phosphineylphosphine, and Zn_2_(PdTCPP)(DPDS) is decomposed into monolayers of MOF nanosheets (Figure 4d).

The top‐down approach is simple, but has a low yield, which makes it more difficult to use practically. Therefore, finding a more effective stripping method is necessary to increase the yield.^[^ ^87^ ^]^

#### 3.1.2 Bottom‐Up Method

**Figure 5:**
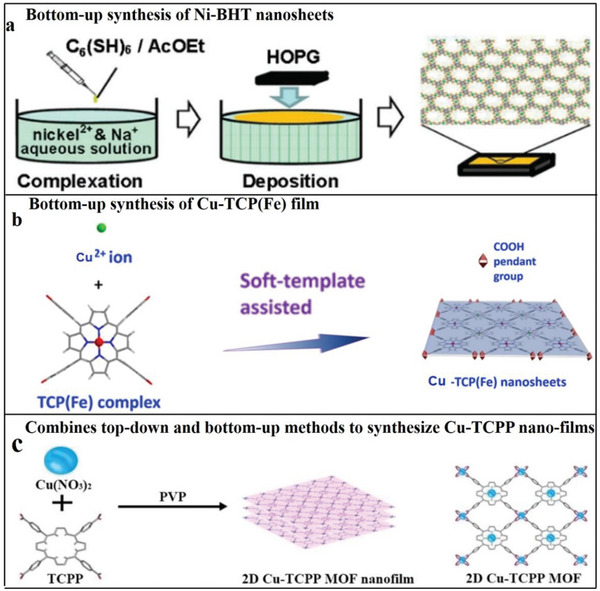
a) Principle scheme: f Ni‐BHT nanosheet synthesis at water–air interface. Reproduced with permission.^[^ ^97^ ^]^ Copyright 2013, American Chemical Society. b) Synthesis graph of polycation‐regulated 2D Cu‐TCP(Fe) membrane. Reproduced with permission.^[^ ^99^ ^]^ Copyright 2017, American Chemical Society. c) Framework of Cu‐TCPP nanofilm and synthesis diagram of 2D Cu‐TCPP nanofilm. Reproduced with permission.^[^ ^23^ ^]^ Copyright 2019, Royal Society of Chemistry.

The bottom‐up medium is used to synthesize 2D MOFs nanoflakes from organic ligands and metal ions. This method inhibits the germination of MOF in one direction to form 2D MOFs. Zhu et al. synthesized Cu‐BHT nanosheets at the water–dichloromethane junction surface using a bottom‐up approach.^[^ ^96^ ^]^ BHT was added to dichloromethane, and water was added to the admixture to form an oil–water junction surface. Cu and BHT ligands meet at the interface to constitute Cu‐BHT nanosheets. Nishihara et al. synthesized Ni‐BHT nanosheets by the bottom‐up method.^[^ ^97^, ^98^ ^]^ They inserted the BHT ligand into the ethyl acetate liquor, and added the mixture into aqueous solution. Afterwards, the ethyl acetate was evaporated, and a stratum of BHT ligands was formed on the water skin layer. After 2 h, Ni‐BHT nanocrystal was formed on the liquid‐gas junction surface, and turned into the highly oriented pyrolytic graphite substrate (**Figure** **5**a).

When studying 2D MOFs, Ang et al. synthesized a 2D Cu‐TCP(Fe) membrane by the bottom‐up approach. First, Cu‐TCP(Fe) nanomaterials were made by a soft template‐assisted method comprising Cu^2+^ and TCP (Fe) complexes, forming a leachy framework. (Figure 5b).^[^ ^99^ ^]^ Using the method of preparing the Cu‐TCP (Fe) membrane, Ang and Hong synthesized the Co‐TCP(Fe) and Zn‐TCP(Fe) membranes by replacing Cu bits with Co and Zn bits, respectively.^[^ ^99^ ^]^ He et al. synthesized a 2D‐ordered hole IRMOF‐8 (H‐IRMOF‐8) material while studying 2D MOFs. The synthesis method is relatively simple. DMAC was added to a flask, and Zn(OAc)_2_·2H_2_O, 2,6‐naphthalene dicarboxylic acid, lanthanum and PVP k‐30 were added to the DMAC. The compound was magnetically stirred at 60 °C for 30 min, separated by centrifugation, and purified with ethanol many times. During the synthesis, the mesopores were introduced into the original IRMOF‐8 via a competitive coordination strategy.^[^ ^25^ ^]^ Jiang et al. proposed 2D hierarchically porous carbon (2D‐HPC) nanosheets, which can be applied as the main material in lithium–sulfur batteries. Jiang et al. synthesized monocline ZIF‐8 nanosheets (at room temperature) and further derived 2D‐HPC nanoflakes (ZIF‐8 nanosheet carbon is expressed as ZIF‐8‐NS‐C). The experimental steps are described as follows: Zn(NO_3_)_2_•6H_2_O and 2‐methylimidazole (Aladdin) were added to two clean measuring glasses of deionized (DI) water and stirred for 24 h at normal temperature to become a white solid. The white solid was collected, cleaned and dried to form ZIF‐8 nanosheets (ZIF‐8‐NS) or ZIF‐8 particles (ZIF‐8‐P). Next, the ZIF‐8 nanoflakes or particles were set on a ceramic bowl and heated to 920 °C under argon flow for 2 h. When the product was cooled, it was mixed with potassium hydroxide at a quality rate of 1:4. The compound was heated to 700 °C for 2 h, and then the temperature of the solution was down in an argon gas stream. The products obtained afterwards were rinsed with water and methanol severally and desiccated at 60 °C for a day to obtain ZIF‐8‐NS‐C (ZIF‐8‐P‐C). Next, sulfur was added at a mass ratio of 3:7, and was heated at 155 °C for 20 h. Furthermore, it was cooled by argon flow, and the resulting product was named S/ZIF‐8‐NS‐C (S/ZIF‐8‐P‐C).^[^ ^28^ ^]^

2D ZIF materials have strong adsorption capacities, so researchers aim to synthesize ZIF materials to be used as adsorbents to remove heavy metals, fuels and other harmful substances.^[^ ^25^, ^100^, ^101^, ^102^ ^]^ Nasir et al. synthesized 2D leaf‐shaped ZIF (ZIF‐L) using the top‐down approach as an adsorbent. The synthesis method is as follows.^[^ ^25^, ^101^ ^]^ First, hexahydrate zinc nitrate (Zn(NO_3_)_2_·6H_2_O) and dimethyl sulfoxide were added to DI water. The resulting mixture was stirred for 4 h. After centrifugation for another 10 min, the resulting product was rinsed thrice with fresh methanol and desiccated in the cabinet for a night.^[^ ^25^ ^]^ RiouCavellec et al. synthesized 2D trimer iron [Fe(H_2_O)_2_(C_9_O_6_H_4_)]H_2_O via the hydrothermal method, which is a bottom‐up approach, and named it MIL‐67. MIL‐67 was synthesized by mixing iron powder, 1,2,4‐benzene tricarboxylic (or terephthalic) acid and water. Afterwards, the mixture was added to a PTFE‐lined stainless steel acid digester and heated. The mixture was filtered and the resulting solids were rinsed with distilled water and desiccated.^[^ ^103^ ^]^ Serre et al. synthesized 2D [Ti_3_O_2_(OH)_2_(HPO_4_)_2_(PO_4_)_2_]·(NH_3_‐(CH_2_)_3_‐NH_3_)_2_·(H_2_O)_2_ through a bottom‐up approach and named it MIL‐28_3_.^[^ ^104^ ^]^ MIL‐28_3_ is of the 2D structure. Hydrated titanium dioxide, H_3_PO_4_, HF, 1,3‐diamino propane and H_2_O were mixed. By placing the mixture in a Teflon‐lined steel autoclave and leaving it to rest for 4 d, a mixture with pH = 4 can be formed. The resulting white sediment was washed with softened water and dried at room temperature.

#### 3.1.3 Combination of Top‐Down and Bottom‐Up Methods

**Figure 6:**
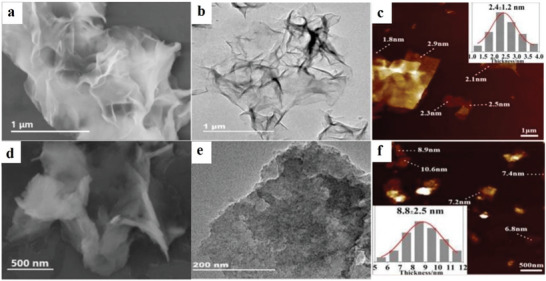
a–c) SEM, TEM and AFM images of 2D Cu‐TCPP nanofilm. d–f) SEM, TEM and AFM images of 2D Cu‐TCPP nanosheet. Reproduced with permission.^[^ ^23^ ^]^ Copyright 2019, Royal Society of Chemistry.

Some studies combine top‐down and bottom‐up approaches to synthesize 2D MOFs. Bai et al. synthesized 2D MOFs Cu‐TCPP nanofilms in the presence of PVP.^[^ ^23^ ^]^ The main experimental process entailed preparing the material using a top‐down approach and the auxiliary action of surfactants. Cu was used as the ligand ion, and tetra(4‐carboxyphenyl) porphyrin (TCPP) was the ligand. The preparation process is shown in Figure 5c. A TCPP molecule is connected with four Cu metal nodes of impellers, which can form a layered crystal structure in the form of accumulation in the presence of PVP, thus forming 2D MOF Cu‐TCPP film. The samples synthesized by the top‐down method have a good dispersion structure and high yield.^[^ ^25^, ^105^, ^106^, ^107^, ^108^ ^]^ The thickness of the obtained 2D MOF Cu‐TCPP film mentioned above is 1–3 nm, which is rare in the reported structures of 2D MOFs. The obtained MOF Cu‐TCPP nanofilms were examined using scanning electron microscopy (SEM), transmission electron microscopy (TEM) and atomic force microscopy (AFM), and **Figure** **6**a–c were obtained. Evidently, the maximum thickness of Cu‐TCPP nanofilm is merely 2 nm, the size is several hundred nanometers to several microns and the structure is folded. Bai et al. also synthesized Cu‐TCPP nanoflakes in the presence of PVP through the same experimental process. The thickest of the synthesized Cu‐TCPP nanosheet is only 6–10 nm, which is a good thickness in reported 2D MOF materials.^[^ ^23^ ^]^ Lu et al. synthesized 2D MOF nanosheets by applying the top‐down approach and the surfactant‐assisted growth of Au nanoparticles, but the thicknesses of the nanosheets were relatively thick, ≈20 nm. The thickness of iron porphyrin MOF nanoparticles synthesized by Zhang et al. is thinner than that of Lu et al., about 3–7 nm. The thickness of the majority of 2D MOF nanomaterials exceeds 10 nm, and a small amount of material below 10 nm can be made.^[^ ^23^ ^]^ The obtained MOF Cu‐TCPP nanometer sheet was investigated using SEM, TEM and AFM, and Figure 6d–f was obtained. Evidently, the Cu‐TCPP nanometer comprises some anomalous 2D thin sheets with a thickness of ≈8 nm and a size of dozens to hundreds of nanometers. Bai et al. prepared 2D MOF Co‐TCPP nanofilms and Co‐TCPP nanoflakes, respectively, in the absence and presence of PVP by the above experimental method (replacing the Cu bits with Co bits).^[^ ^23^ ^]^

### 3.2 COFs

In recent years, COFs have attracted great interest,^[^ ^109^, ^110^, ^111^, ^112^ ^]^ and several methods have been explored to synthesize COFs, such as the solution method, microwave heating,^[^ ^113^, ^114^, ^115^, ^116^ ^]^ and ion thermal method.^[^ ^48^, ^117^ ^]^ Herein, each method will be discussed in detail.

#### 3.2.1 Sonochemical Synthesis Method

**Figure 7:**
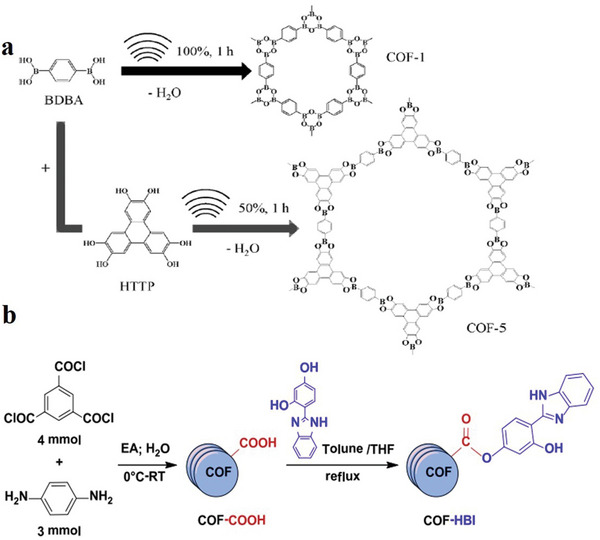
a) Synthesis of COF‐1 and COF‐5 is accomplished by sonochemical processes. Reproduced with permission.^[^ ^120^ ^]^ Copyright 2012, the Royal Society of Chemistry. b) Description of COF‐COOH and COF‐HBI. Reproduced with permission.^[^ ^121^ ^]^ Copyright 2014, Elsevier Inc.

Sonochemical is another procedure that results in the production of small MOF crystals with less reaction time. In this process, the crystallization rate rapidly increases owing to the bursting and formation of bubbles in the solution, which is called acoustic cavitation,^[^ ^118^, ^119^ ^]^ which generates a high temperature (5000 K) and pressure, leading to rapid heating and cooling rates. In previous studies, the synthesis of 2D COFs was mainly conducted using the sonochemical synthesis method. Juan et al. synthesized COF‐1 and COF‐5 by the sonochemical synthesis method: 1,4‐Benzenediboronic acid (BDBA) and 2,3,6,7,10,11‐hexahydroxybiphenyl (HHTP) is a kind of 2D skeleton COF‐5. The B_3_O_3_ (boroxine) ring formed by the dehydration reaction of a single BDBA molecule with a planar Hexa‐hydroxyl 2D skeleton is called COF‐1. The operability of the sonochemical synthesis of the COF structure is proved by this process (**Figure** **7**a). Although sonochemical synthesis is a relatively new method, which assists in decreasing the large reaction times and high temperatures, it needs further exploration for better understanding.^[^ ^120^ ^]^

#### 3.2.2 Solution Method

The solution method is the most common method used for material synthesis that separates the precipitate from the solution. The study of Juan et al. is based on the solution method. In Figure 7b, the preparation method of solid‐phase extractant is shown, which is explained in three steps as presented below: COF matrix preparation, HBI (2‐(2,4‐hydroxyphenyl)‐benzimidazole) synthesis and functionalization. First, ethyl acetate (EA) and trimethyl chloride were used to prepare a transparent and colorless solvent. Next, o‐phenylenediamine was dissolved in a solvent (using Na_2_S_2_O_5_ as a catalyst), and vacuum filtration was performed to obtain a solid product.^[^ ^122^ ^]^ Then, a mixed solvent of CHCl_3_ and CH_3_OH was used for recrystallization to obtain yellow crystals. Finally, a COF‐based solid‐phase extractant was prepared to obtain COF‐HBI.

#### 3.2.3 Ionothermal Synthesis

The study of Sophie et al. used the ionothermal synthesis of an acid‐catalyzed triazine network (pre‐CTF). First, the appropriate ratio of CHCl_3_ and tri‐fluorosulfonate was put into a dry round bottom flask in an inert atmosphere. When the mixture changed from colorless to yellow, a solid precipitate appeared. Afterwards, it was cooled to room temperature, and an appropriate amount of DI water containing 25% ammonia was added. The solid precipitate was vacuum filtered, and the impurities were washed.

The synthesis of CTF‐1 was performed in a crucible. The pre‐CTF and ZnCl_2_ mixture was placed in a crucible in an appropriate ratio, and it was stored in an enclosed space of inert Rn gas. Next, the mixture was placed in an argon furnace that had been preheated for 30 min. The crude product was ground, and DI water was added to wash excess impurities repeatedly.

#### 3.2.4 Microwave Synthesis

Nowadays, most researchers use atmospheric pressure microwave high‐temperature ion heating to prepare CTF‐based magnetic composite materials. During the experiment, according to different reaction conditions, an appropriate amount of ferric chloride hexahydrate (FeCl_3_·_6_H_2_O) is dissolved in ethanol, 1,4‐dicyanobenzene (DCB) and anhydrous zinc chloride (ZnCl_2_) to form a mixed solution, and was put it into a crucible. Finally, the crucible is placed in the microwave oven for the experiment.

### 3.3 HOFs

Nowadays, most HOFs are synthesized by solvent evaporation, diffusion and solvothermal synthesis. However, each method has its advantages and inevitable defects. There are different synthesis methods used to produce HOFs materials with different structures; some are given below.

#### 3.3.1 Solvent Evaporation Method

The so‐called solvent volatilization method dissolves the organic ligand in a small beaker in an appropriate solvent according to a certain proportion, and crystallizes the framework through the slow volatilization of the solvent.^[^ ^66^, ^123^, ^124^, ^125^, ^126^ ^]^ To grow a single crystal with better crystal form, filter paper or plastic film is used to slow down the volatilization speed (poking 3–5 small holes with needles). The operating principle of this method is relatively simple, and it is relatively easy to obtain the product with better crystal form and larger particles. However, it must be performed at a static room temperature, usually using a solvent with a lower boiling point, and generally require a longer reaction time. Luo et al. reported a super stable 2D microporous organic framework material (HOF‐8), which was assembled using an amide pyridine compound containing a hydrogen bond donor and acceptor in the structure.^[^ ^123^ ^]^ In 2017, Hong took 3,3′,5,5′‐tetrakis‐(4‐carboxyphenyl)‐1,1′‐biphenyl (H4TCBP) as the ligand, dissolved it in N‐N‐N‐di‐methyl‐formamide and left the solution open at room temperature to volatilize slowly. After a few days, the hydrogen‐bonded organic skeleton material HOF‐TCBP with ultra‐high thermal stability was obtained.^[^ ^66^ ^]^

#### 3.3.2 Diffusion Method

##### Vapor Diffusion Method

The organic ligand is dissolved in a benign solvent with a high boiling point and volatilized. Then, another low‐volatile solvent (such as triethylamine, pyridine and acetone) is diffused into the former solution in a closed container to reduce the solubility of the solute and precipitate the framework. The most generally used method is the big‐bottle and small‐bottle method, and the solvents chosen must be mutually dissolved.^[^ ^59^, ^127^ ^]^ In 2011, Chen's research group used the steam diffusion method to construct the first hydrogen‐bonded organic skeleton material (HOF‐1) with a stable structure.^[^ ^59^ ^]^

##### Liquid‐Phase Diffusion Method

The reactants are diffused through the solvent near the interface, and another solvent is added in the middle to slow down the diffusion rate. This method of operation is based on the low solubility of the reactants in the solvent, which slows the reaction and obtains better crystal quality and morphology.^[^ ^128^, ^129^ ^]^ Nugent et al. synthesized 2D MPM‐1‐TIFSIX by the liquid‐phase diffusion method. At room temperature, adenine dissolved in acetonitrile/H_2_O was placed above the aqueous solution containing Cu(NO_3_)_2_·2.5H_2_O and (NH_4_)_2_TiF_6_, and acetonitrile/H_2_O was placed in the layer between the top and bottom solutions to slow down the reaction rate. After 4 d, purple rectangular prisms were formed.^[^ ^128^ ^]^

#### 3.3.3 Solvothermal Synthesis

Solvothermal synthesis cultivates high‐quality crystals under relatively harsh conditions. Some organic ligands that are insoluble or insoluble at normal temperatures have increased solubility under the high temperature and pressure of the solvothermal reaction, which is beneficial to the reaction.^[^ ^125^, ^130^ ^]^ The reaction process is often accompanied by some chemical reactions that are difficult to occur under normal conditions, making it possible for solvents to extract solid components and crystal growth. Choosing the appropriate solvent is conducive to obtaining well‐oriented and perfect crystals.^[^ ^131^, ^132^, ^133^ ^]^ In solvothermal reactions, the commonly used solvents are dimethyl sulfoxide (DMSO), N‐methylpyrrolidone (NMP), dimethylformamide (DMF), dimethylacetamide (DMA) and anhydrous ethanol.^[^ ^70^, ^125^, ^134^, ^135^ ^]^ In 2013, Yoon et al. prepared 2D HOF‐BTB with unimodal 3‐c honeycomb (HCB) topology by the solvothermal synthesis.^[^ ^134^ ^]^ Excess H_3_BTB was dissolved completely in a small amount of DMF in a hot oven and cooled in a refrigerator. The obtained crystals were collected and dried in the air and immersed in methanol. The methanol solution was left standing in an oven to obtain a highly strong micro‐porous hydrogen‐bonded 2D organic skeleton material HOF‐BTB. In 2014, Miljanic et al. prepared 2D HOF materials with stable structures by solvothermal synthesis.^[^ ^130^ ^]^ The 2D HOF materials with light weight and stable heat and hydrolysis were obtained by dissolving fluoropyrazole molecules as ligands in the mixed solution of N‐N‐N‐di‐methyl‐formamide and methanol and heated at 80 °C for a day.

### 3.4 Perovskite Synthesis

Depending on the source material (2D or 3D perovskite), different methods can be used to process the material. First, the perovskite raw material is formulated into a precursor solution, and the solvent is evaporated to obtain an excellent single‐crystal thin film. Next, the mechanical exfoliation method can also be used to peel the 2D organic perovskite material of the van der Waals stack structure into a 2D Ruddlesden–Popper perovskites (RPP) with a single quantum well thickness. Additionally, the spin‐coating technology method is the most common film synthesis method, which can help reduce the film thickness.

#### 3.4.1 Solution Methods

**Figure 8:**
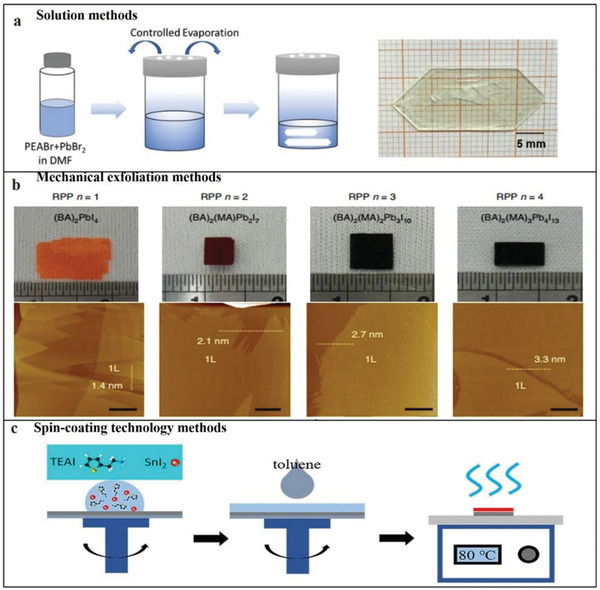
a) Schematic of growing (PEA)_2_PbBr_4_ single crystal using solution method. Reproduced with permission.^[^ ^136^ ^]^ Copyright 2019, Royal Society of Chemistry. b) Photographs of centimeter‐sized RPP single crystals and AFM images after mechanical peeling. Reproduced with permission.^[^ ^138^ ^]^ Copyright 2018, Nature Publishing Group. c) Schematic of manufacturing process of 2D organic perovskite thin‐film spin‐coating process. Reproduced with permission.^[^ ^139^ ^]^ Copyright 2020, American Chemical Society.

In previous studies, 2D organic perovskite materials were mainly synthesized using solution methods. Zhang et al. developed a solvent evaporation technique controlled at a constant temperature to obtain the best single‐crystal quality.^[^ ^136^ ^]^ First, the three substances (DMF, PEABr and PbBr_2_) were fully dissolved at 23 ± 0.5 °C to form a (PEA)_2_PbBr_4_ precursor solution, and filtered to produce a clear solution. Then, a closed plastic container was used to hold 6 mL of the precursor solution, and a hole punch was used to design an array of two openings with a 1 mm diameter on the plastic container. Finally, the container was placed in an oven (23 ± 0.5 °C) to grow for ≈20 d, thereby obtaining a centimeter‐level (PEA)_2_PbBr_4_ single crystal (**Figure** **8**a). Also, Cohen et al. synthesized 1,4‐benzenedimethanamonium iodide in an ice bath.^[^ ^137^ ^]^ After adding the acid, it was left to stand for 20 min to produce a precipitate. The precipitate was washed with ether and anhydrous ethanol to recrystallize the precipitate. Next, the solution was added to the perovskite solution mixture by controlling the stoichiometric ratio to successfully prepare the (BzDA)A_9_Pb_10_ 2D perovskite solution. The solution synthesis method is the most effective method to synthesize 2D organic perovskites.

#### 3.4.2 Mechanical Exfoliation

Mechanical exfoliation is also an effective method for obtaining perovskite thin films by exfoliating bulk RPP crystals between organic layers. Leng et al. studied a temperature‐programmed solution precipitation method, and the perovskite material was successfully prepared using this method.^[^ ^138^ ^]^ 2D lead Ruddlesden–Popper perovskites sing crystals were successfully synthesized using three solid precursors: PbO, BAI, and MAI. Then, the thin molecular layer of large molecules was peeled from the single crystal (Figure 8b).

#### 3.4.3 Spin‐Coating Technology

The spin‐coating process results in the production of smooth and dense films. Wang et al. reported using spin‐coating technology to obtain good PEA_2_SnI_4_ and TEA_2_SnI_4_ films (Figure 8c).^[^ ^139^ ^]^ The mixed solution of PEAI (or TEAI) and SnI_2_ were dissolved in DMF and DMSO to obtain the perovskite precursor solution. According to previous reports, the perovskite has no excellent film‐forming ability. Therefore, Wang et al. prepared a dense film using toluene to obtain smoother and denser PEA_2_SnI_4_, and DMF was used as a solvent to obtain a good TEA_2_SnI_4_ film.^[^ ^139^ ^]^

Through these methods, 2D organic perovskite materials can be successfully synthesized. However, so far, the obtained film thickness still has a gap in practical applications, so achieving a thinner film remains a challenge.

### 3.5 Molecular Crystals

Self‐assembled 2D molecular crystal materials with weak van der Waals bonds have a single‐layer or multilayer molecular structure.^[^ ^141^ ^]^ By using the solution preparation technology, the high precision and large‐size 2D molecular crystal films can be conveniently and efficiently prepared. Currently, the main solution technologies are drop casting, spin coating, dip coating and many other different methods used to prepare different 2D molecular crystals.

#### 3.5.1 Solution‐Immersion Assembly

The solution‐immersion assembly technology is mainly a technology that spontaneously forms organized nanostructures or patterns in functional, structural components, which is mainly driven by a stable, uniform and nondirectional solvent evaporation process. The thickness of the produced 2D molecular crystal reaches the nanometer level, forming a thin film or a single‐crystal semiconductor.

**Figure 9:**
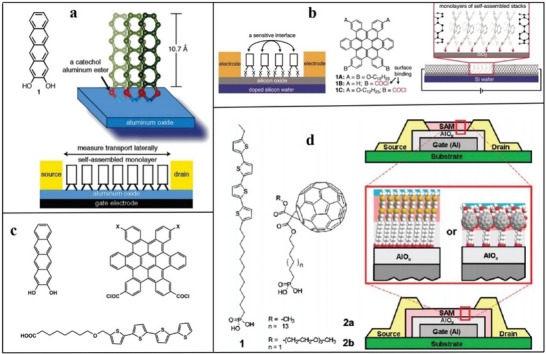
Solution‐immersion assembly technique. a) Reproduced with permission.^[^ ^142^ ^]^ Copyright 2014, American Chemical Society. b) Reproduced with permission.^[^ ^143^ ^]^ Copyright 2006, American Chemical Society. c) Reproduced with permission.^[^ ^144^ ^]^ Copyright 2010. d) Probing dynamic interfaces in organic electronics. Reproduced with permission.^[^ ^145^ ^]^ Copyright 2011, American Chemical Society.

In 2004, Tulevski et al. studied a new tetraene derivative that can form a dense, upright monolayer film on the surface of alumina.^[^ ^142^ ^]^ This new tetraene derivative single‐layer film can spontaneously self‐organize to form the active layer in nano‐field effect transistor devices (**Figure** **9**a). In 2006, Guo et al. reported a unique single‐molecule layer of polycyclic aromatic hydrocarbons with multifunctional molecules, which are assembled laterally into pillars and attached to the silicon oxide surface of silicon wafers.^[^ ^143^ ^]^ An effective transistor comprises many single molecules; when the molecules lacking electrons are exposed, the electrical properties will change significantly, thus creating a new environment (Figure 9b). In 2010, Mathijssen et al. synthesized a semiconductor molecule in which pentathiophene and monochlorosilane were the core and anchor groups.^[^ ^144^ ^]^ Solution‐submerged assembly organic field‐effect transistors (OFETs) were prepared by the liquid crystal molecular method, and the test device results revealed that the carrier structure does affect the channel length (Figure 9c). With technological development, in 2011, Novak et al. synthesized multifunctional molecules with self‐assembly capabilities based on tetra‐thiophene and fullerene units. Based on tetra‐thiophene and fullerene units, multifunctional materials with self‐assembling abilities were synthesized.^[^ ^145^ ^]^ This research enabled the low‐voltage operation of self‐assembled single‐layer transistors (Figure 9d).

#### 3.5.2 Drop‐Casting and Spin‐Coating

**Figure 10:**
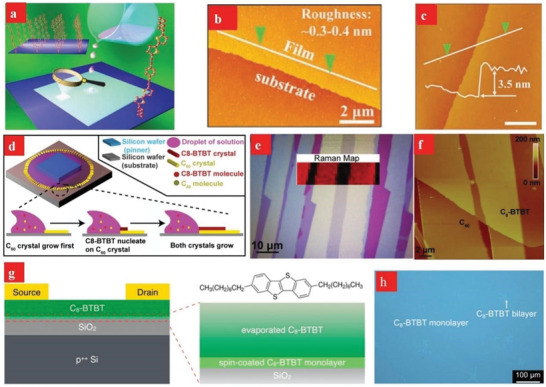
a–f) Drop‐casting technique for fabricating HTEB and C_8_‐BTBT crystals, respectively. a–c) Reproduced with permission.^[^ ^146^ ^]^ Copyright 2011, Wiley‐VCH. d–f) Reproduced with permission.^[^ ^147^ ^]^ Copyright 2013, Wiley‐VCH. g,h) Spin‐casting technique for fabricating C_8_‐BTBT monolayer. Reproduced with permission.^[^ ^148^ ^]^ Copyright 2018, American Chemical Society.

Drop‐casting methods and spin‐coating methods are common solution treatment methods. Among them, the drop‐casting method is commonly used to deposit organic semiconductor crystal films. In 2011, Jiang et al. used drop‐casting to successfully prepare 2D crystals of organic semiconductors on the millimeter scale for the first time.^[^ ^146^ ^]^ First, the chlorobenzene solution on different substrates (e.g., SiO_2_, quartz and even other amorphous substrates) cast millimeter‐sized 2D 1,4‐bis((5ʹ‐hexyl‐2,2 ʹ ‐bithiophen‐5‐yl)ethynyl)benzene (HTEB) crystal films (**Figure** **10**a). The film prepared via drop‐casting was very smooth and had a perfect structure. More importantly, through TEM images and selected area electron diffraction patterns, it can be found that high‐quality 2D films have superior electrical properties (Figure 10b,c), which effectively confirms that the number of layers of 2D materials affects the charge carrier mobility. In addition, Fan et al. developed a droplet‐pinned crystallization method for preparing organic single‐crystal p–n junctions from solutions of organic semiconductor mixtures (Figure 10d–f).^[^ ^147^ ^]^ Drop‐casting greatly improves the mobility of electrons, provides a platform for single‐crystal p–n junctions and provides a good foundation for manufacturing high‐performance electronic devices of organic semiconductors.

To better reflect the inherent advantages of uniform deposition and large‐area molecular films, Wang et al. developed a technique involving antisolvent crystallization to grow spin‐coating monolayer molecular Ar crystals (Figure 10g,h).^[^ ^148^ ^]^ The spin coating method is always used to deposit an aligned dioctylbenzothienobenzothiophene (C_8_‐BTBT) single layer on an SiO_2_ substrate. This study proves that a thin, smooth, single‐layer film deposited on the dielectric layer by spin coating can grow a highly ordered upper layer.

#### 3.5.3 Self‐Assembly Method

The epitaxial growth of 2D molecular crystals on functional substrates is very attractive and challenging. Because of the low surface roughness and strong diffusivity of water and air, its assembled 2D molecular crystals can be effectively transferred to the required substrate. This method has attracted special attention. Recently, the solution epitaxial growth technique has been widely used.

**Figure 11:**
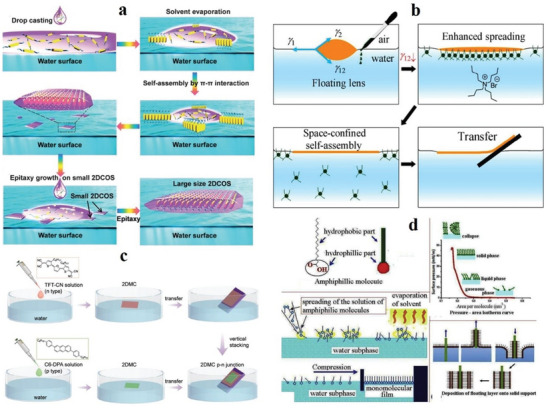
a) Schematic of growing 2DCOS. Reproduced with permission.^[^ ^150^ ^]^ Copyright 2016, Wiley‐VCH. b) Sketches of SCS method. Reproduced with permission.^[^ ^151^ ^]^ Copyright 2018, American Chemical Society. c) Preparation of 2DMC bilayer p–n junctions. Reproduced with permission.^[^ ^152^ ^]^ Copyright 2019, Wiley‐VCH. d) LB technique. Reproduced with permission.^[^ ^157^ ^]^ Copyright 2018, Elsevier Ltd.

The solution epitaxial growth method effectively achieves the epitaxial growth of 2D molecular crystals. Two commonly used technologies are molecular beam epitaxy and physical vapor transport.^[^ ^149^ ^]^ The 2D molecular crystal prepared by Xu et al. using the solution epitaxy method has a large surface area and high quality.^[^ ^150^ ^]^ It includes two steps: i) self‐assembly of micronized 2D molecular crystals on the water surface, and ii) epitaxial growth into 2D molecular crystals with a thickness of several millimeters or centimeters (**Figure** **11**a).

In 2018, Wang et al. used the space‐confined self‐assembly (SCS) method to produce centimeter‐sized 2D molecular crystals.^[^ ^151^ ^]^ The two key steps of the preparation method are i) using DI water as the liquid substrate to minimize the core density, and ii) enhancing the diffusion of the solution on the water surface. A spatialized 2D crystal growth mode is shown in Figure 11b. In 2019, Zhu et al. reported bipolar OFETs based on the two‐layer p–n junction of 2D molecular crystals, which offers high performance and good balance.^[^ ^152^ ^]^ They used the solution epitaxy method to synthesize 2,6‐bis (4‐hexyl phenyl) anthracene (C_6_‐DPA)^[^ ^150^, ^151^, ^153^ ^]^ and a furan–thiophene‐based quinoidal compound (TFT‐CN).^[^ ^154^, ^155^ ^]^ The specific steps are shown in Figure 11c. This type of unipolar semiconductor is based on thin 2D molecular crystals with double‐layer p–n junction, and a bipolar OFET with high performance and good balance is constructed.

#### 3.5.4 Langmuir–Blodgett (LB)

The LB technique has a greater advantage for single‐layer or few‐layer molecular crystals.^[^ ^156^, ^157^ ^]^ LB‐compatible materials have a hydrophilic head group and a hydrophobic tail group. This feature gives the material a unique advantage in synthesizing molecular crystals (Figure 10d). Here, various LB parameters can be controlled to change the deposition scheme. Therefore, LB technology provides a way to connect the macroscopic effects for molecular‐scale control and manipulation. So far, LB films have been widely used in microelectronics and optoelectronics.

#### 3.5.5 Dip and Bar Coating

**Figure 12:**
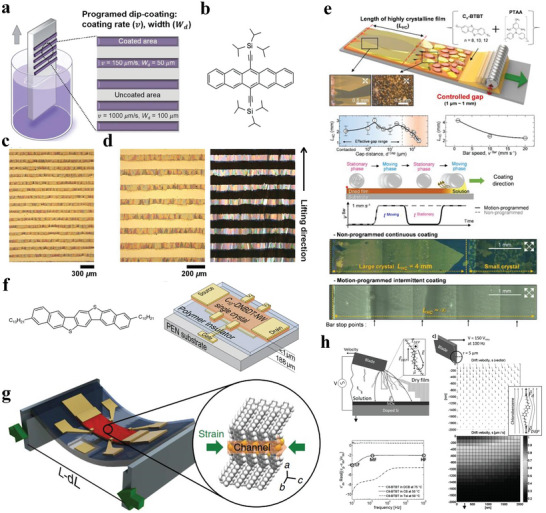
a–d) Programmed dip‐coating process. Reproduced with permission.^[^ ^158^ ^]^ Copyright 2017, Royal Society of Chemistry. e) Wire‐bar coating. Reproduced with permission.^[^ ^159^ ^]^ Copyright 2019, American Chemical Society. f,g) Edge‐casting technique. Reproduced with permission.^[^ ^160^ ^]^ Copyright 2016, Nature publishing group. (h) Application of dielectrophoresis to solution shearing C8‐BTBT. Reproduced with permission.^[^ ^161^ ^]^ Copyright 2017, WILEY‐VCH.

Meniscus‐guided unidirectional coating strategies are mainly used to precisely control morphological carrier transport activities. Dip‐coating can uniquely take advantage of the meniscus and the sliding rod movement of aluminum to pattern the coating material over a large area so that organic semiconductor crystals can grow on curved surfaces. Consequently, it has attracted the attention of many researchers. Nam et al. used a new programmed dip‐coating technique to directly print stripes of highly crystalline soluble propylene crystals.^[^ ^158^ ^]^ **Figure** **12**a,b shows the specific flow of the dip‐coating process. The 6,13‐bis(tri‐isopropylsilylethynyl) pentacene (TIPS‐PEN) crystal successfully forms a pattern on the substrate in the form of micro‐strips with a clear gap. The width (*W*_d_) and gap of the TIPS‐PEN pattern are almost the same as the *W*_d_ programming values of the coated (50 µm) and non‐coated areas (100 µm) (Figure 12c,d).

Bar coating is an effective and attractive technology for preparing highly uniform molecular films with low‐cost, high‐throughput manufacturing roll‐coating technology.^[^ ^132^ ^]^ Lee et al. used gap‐controlled motion to program the bar‐coating layer method and mixed organic semiconductor inks, carefully adjusted the gap and the rod length, extended the crystalline area at the front end of the film and obtained a continuously connected high crystalline C_8_‐BTBT‐PTAA film (Figure 12e).^[^ ^159^ ^]^ This study confirmed the existence of anisotropic crystal structures and polymorphisms with dense unit cells, high uniformity of the strain microstructure and excellent electrical properties. It provides a good idea for promoting the commercialization of organic materials based on next‐generation electronics. Edge‐casting and solution‐shearing are also commonly used techniques. To obtain large‐area single‐crystal thin films, Kubo et al. used edge‐casting to prepare molecular crystals (Figure 12f).^[^ ^160^ ^]^ In this method, the solution is applied between the shear blade and the substrate, and the solution evaporates to form a crystal film. Molina‐Lopez et al. used the solution shearing technology to prepare C_8_‐BTBT (Figure 12h).^[^ ^161^ ^]^ Shearing the blade at the top moves the solution, leaving the meniscus of the solution, and when the solution evaporates, organic molecular crystals are formed.

#### 3.5.6 Floating‐Coffee‐Ring‐Driven Assembly Technology and Pen Writing

**Figure 13:**
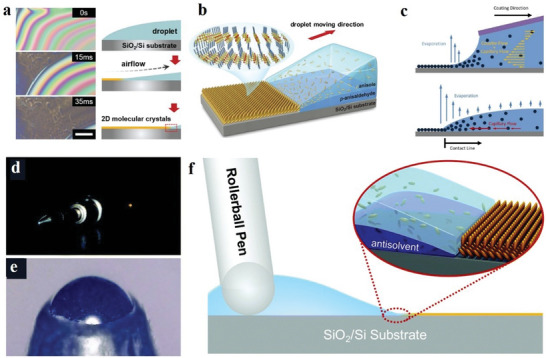
a,b) Schematic of floating‐coffee‐ring‐driven assembly method. Reproduced with permission.^[^ ^162^ ^]^ Copyright 2016, Wiley‐VCH. c) Schematic of unidirectional coating driven by evaporation and droplet undergoing coffee ring effect. Reproduced with permission.^[^ ^163^ ^]^ Copyright 2016, American Chemical Society. d–f) Pen‐writing technology. Reproduced with permission.^[^ ^164^ ^]^ Copyright 2017, the Royal Society of Chemistry.

In addition to the several preparation techniques mentioned above, there are several other methods.^[^ ^162^, ^163^, ^164^ ^]^ 2D molecular crystals prepared by the fluid flow assisted crystallization technology have the advantages of good control of domain arrangement and substrate coverage. This technology controls the morphology of the film to a certain extent. In 2016, Wang et al. prepared a 2D molecular single‐crystal semiconductor C_8_‐BTBT with precise layer defects using floating‐coffee‐ring‐driven assembly technology, laying the foundation for preparing large‐area, low‐cost and high‐performance 2D molecular crystals. In this preparation method, first the solution is dropped on the substrate, and a mechanical pump is used to drag the solution around to form 2D molecular crystals (**Figure** **13**a,b).^[^ ^162^ ^]^ In the same year, Qu et al. used the Marangoni flow‐directed crystal growth to prepare molecular crystals, enhancing nucleation and patterning and easing crystal growth defects. (Figure 13c).^[^ ^163^ ^]^ Pen‐writing technology is also commonly used to prepare 2D molecular crystals in large quantities. Zhang et al. used this technology to prepare 2D molecular crystals with large areas, strong flatness and uniformity. First, the solution is injected into the filled tube, and 2D molecular crystals are manually deposited on the SiO_2_–Si substrate at a stable speed (Figure 13d–f).^[^ ^164^ ^]^

## 4 Properties, Theory and Calculations of 2D Organic Materials

Because of their unique structure and excellent electrical and optical properties, 2D organic materials are the super materials that will lead the future of high‐tech competition.^[^ ^165^, ^166^, ^167^, ^168^ ^]^ Here, the electrical, optical, magnetic and chemical properties of 2D organic materials (MOFs, COFs, HOFs, perovskite and molecular crystals) are explained.

### 4.1 Electronic Properties of 2D Materials

Electrical properties are one of the most basic properties of materials because the atoms and molecules that make up the material are produced by the interaction of electrons. Understanding the electrical properties of materials is greatly significant for the preparation and application of materials.^[^ ^168^, ^169^ ^]^ The electrical properties of each material are described in detail as follows:

#### 4.1.1 Electronic Properties of MOFs

**Figure 14:**
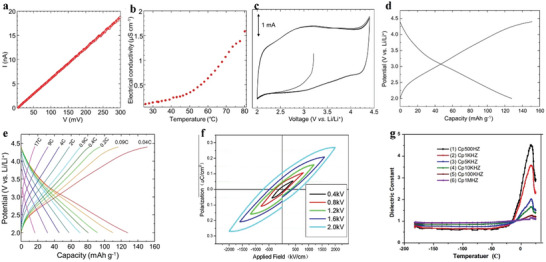
a) *I*–*V* curve of globose Cu‐CuPc measured at room temperature. b) Conductivity of globose Cu‐CuPc varies with temperature in the range of 25 °C–80 °C. c) Cyclic voltammogram of Cu‐CuPc cathode. d) Charge–discharge curve of Cu‐CuPc cathode. e) Charge–discharge curves of Cu‐CuPc cathode at different C rates. Reproduced with permission.^[^ ^170^ ^]^ Copyright 2017, John Wiley and Sons. f) Electromagnetic loop at different frequencies. g) Relationship between temperature and dielectric constant at different frequencies. Reproduced with permission.^[^ ^171^ ^]^ Copyright 2014, Elsevier Science.

Electrical property is an important property of 2D MOF materials. Nagatomi et al. studied the electrical performance of 2D MOF Cu‐CuPc.^[^ ^170^ ^]^ During the experiment, the Cu‐CuPc powder was stressed to spherules, and the *I*–*V* curve of the spherules was sounded with a gold wire double probe. At room temperature, the ohmic resistance ranging from 0 to 300 mV is shown in **Figure** **14**a. The conductivities at a constant voltage (150 mV) and different temperatures are shown in Figure 14b. They mixed Cu‐CuPc, a poly‐tetra‐fluoro‐ethylene (PTFE) adhesive, and carbon black. Next, the mixture was cast into a pollution‐free grid to make the cathode, which was installed on a simple LiB battery device. Afterwards, the cathode capacity was estimated through charge–discharge processes. During the experiment, volt–ampere tests were conducted on Li/Li^+^ at a potential range of 2.0–4.4 V, and wide reduction–oxidation (REDOX) peaks appeared at a scanning speed and voltage of 1 mV s^−1^ and 3.0 V, respectively (Figure 13c). Similarly, the voltage was maintained between 2.0 and 4.4 V, and the current was constant at 0.04 C. After the charge–discharge cycles, the capacitance was undegraded, and the charging and discharging capacities reached 151 and 128 mAh g^−1^, respectively (Figure 13d). The results show that the cathode has a large and stable charging and discharging capacities and cycle, respectively. Low conductivity directly affects the high coulomb rate of the MOF‐based cathode. Figure 14e shows the capacity of Cu‐CuPc at different charging and discharging rates. The electro‐conductibility of Cu‐CuPc plays a significant role in achieving a jarless charge–discharge cycle. Tan et al. researched the ferroelectric performance of a 2D MOF, which was a typical ferroelectric compound.^[^ ^171^ ^]^ They plotted the hysteresis loops of the complex at the voltages of 0.4, 0.8, 1.2, 1.6 and 2.0 kV (Figure 14f). Figure 14f shows the residual polarization (Pr) and the coercive field (Ec) values of 0.029 µC cm^−2^ and 0.4 kV cm^−1^, respectively. Tan et al. also plotted the relationship between the dielectric constant and temperature at different frequencies (Figure 14g).^[^ ^171^ ^]^ Evidently, the permittivity below –21 °C is ≈0.5 and remains constant. When the frequency decreases below 500 Hz, the frequency of the AC electric field greatly influences the permittivity changes with temperature.

#### 4.1.2 Electronic Properties of COFs

2D COFs are a new electroactive material mainly used in electronics and chemical resistance sensing. However, 2D COFs still have unresolved issues, mainly with their structure, chemistry and conductivity.^[^ ^172^ ^]^ Pyridine 2D COFs containing Zn and Cu have been synthesized by condensation reaction. Metal pyridine (M = Zn and Cu) and pyrethroid derivatives were used as raw materials. The specific mobility parameter is 5 cm^2^ (V s)^−1^, which was measured under the DC limit.^[^ ^173^ ^]^ The metal center from copper to zinc in phthalocyanine is very small for related parameters. These parameters are divided into three aspects, namely conductivity (conductivity parameter ≅ 5 × 10^−7^ S cm^−1^), carrier scattering rate (scattering rate ≅3 × 10^13^ s^−1^) and carrier density (density ≅10^12^ cm^−3^). The carrier density has little effect, which is related to the effective mass (≈2.3 mg) of most loads (holes). This conclusion is based on the measurement of terahertz (THz) spectroscopy, Hall effect measurement and density functional theory.^[^ ^167^ ^]^ Note that carrier transport is anisotropic; for this 2D COF, the fluidity of the hole in the plane is zero, which is limited outside the plane.

#### 4.1.3 Electronic Properties of HOFs

Proton conduction is one of the most important characteristics of 2D HOFs. A proton conductor is a kind of electrolyte that conducts electricity. There are two ways of conducting the protons: first, the conduction between the proton conductor and the anion, called the Grotthuss Gr mechanism. Second, the proton conductor conducts electricity with other ions, called the mediation mechanism.^[^ ^174^ ^]^ Because 2D HOF materials have acid–base electron pairs, which can form intramolecular hydrogen bonds with each other, a network structure interacting with hydrogen bonds is formed. Besides, this structure is the channel of proton conduction. 2D HOF materials use hydrogen bonding as one of the main forces connecting organic structural units with electron donors and acceptors, and self‐assembly and dipole–dipole interaction with the help of other weak interactions, such as *π*–*π* and van der Waals interaction.^[^ ^175^ ^]^ Because hydrogen bonds have great freedom, organic structural units can form different hydrogen bond models in different solvents or temperatures, thus obtaining hydrogen‐bonded organic skeleton materials with different structures. Therefore, HOF materials are highly sensitive to solvents.^[^ ^176^ ^]^ In addition, *π*–*π* stacking, CH·*π* interaction and van der Waals force can further enhance the stability of the skeleton structure, and obtain HOF skeleton materials with excellent stability.^[^ ^66^, ^177^, ^178^ ^]^

#### 4.1.4 Electronic Properties of Perovskites

Perovskite materials are widely used in many fields owing to their composition, flexibility and structural variability. The chemical formula of organic perovskite materials is ABX_3_, where A is replaced by organic cations. A, B, and X can be replaced by various elements. Therefore, the material structure can be changed by replacing the position of elements to adjust the bandgap to obtain different electronic properties. Amat et al. studied the perovskite materials of A = FA and MA through first‐principles calculations. They proved that the bandgap change in perovskite materials was induced by replacing the A sites with FA (1.5 eV) and MA (1.55 eV).^[^ ^179^ ^]^ The excellent physical properties of 2D organic perovskite materials are attributed to the rapid migration of ions. Since the ions in the perovskite have low migration activation energy (EA), the Schottky defect promotes ion migration.^[^ ^180^ ^]^ Eames et al. studied the activation energy of ion migration in MAPbI_3_.^[^ ^181^ ^]^ The EA values of MA^+^ and Pb^2+^ ions are 0.84 and 2.31 eV. Since defects are mobile and abnormal defect properties can cause high ionicity of perovskite materials, it will participate in ion migration under external bias. Besides, ion migration is an essential characteristic of perovskite materials. In addition, Fu et al. studied the effect of perovskite cations on the structure and photoelectric properties of the device.^[^ ^182^ ^]^ The tolerance factor in perovskites restricts the number of cations that enter its cage. However, a special cationic, ethylammonium, can be stretched into the cage of 2D halide perovskites. Based on this principle, several cationic‐engineered 2D RPP iodized perovskites were prepared. The single‐crystal structural analysis shows that the Pb‐1 bond stretching can be observed in the perovskite material doped with ethylammonium, which enlarges the cage and causes larger octahedral deformation. This conclusion once again enriches the types of 2D organic perovskites.

#### 4.1.5 Molecular Crystals

**Figure 15:**
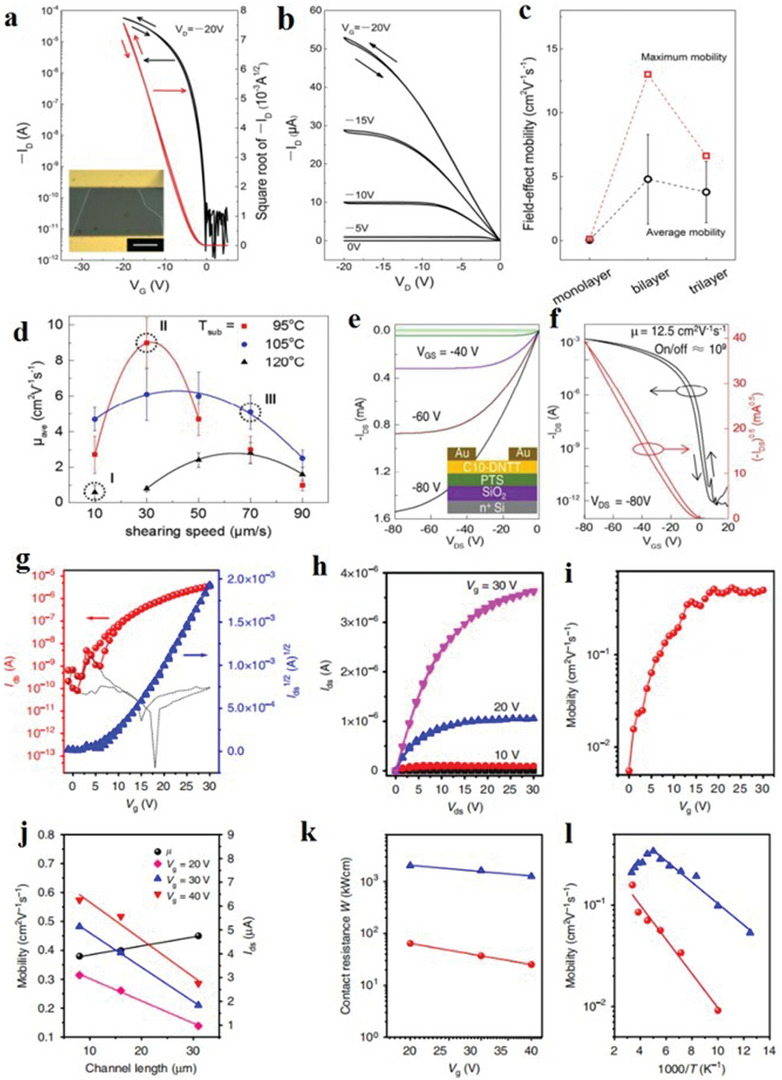
a–c) Transistor characteristics for 2D crystalline C_8_‐BTBT. Reproduced with permission.^[^ ^162^ ^]^ Copyright 2016, Wiley‐VCH. d) Mean values of saturated mobility and standard deviations. e,f) Output and transfer *I*–*V* curves. Inset of (e) is the structure of OFET device. d–f) Reproduced with permission.^[^ ^183^ ^]^ Copyright 2016, the Royal Society of Chemistry. g–l) Electrical properties of MMC FETs. g–l) Reproduced with permission.^[^ ^184^ ^]^ Copyright 2018, Nature publishing group.

Charge carrier transport is an important electrical characteristic that often occurs at the semiconductor dielectric interface. Wang et al. prepared a double‐layer C8‐BTBT crystal film and performed electrical measurements.^[^ ^162^ ^]^ **Figure** **15**a shows the transmission characteristics of the device at a drain voltage of ‐20 V. Evidently, the double‐layer C_8_‐BTBT device has higher field‐effect mobility, which can reach 5.2 cm^2^ V^−1^ s^−1^. Figure 15b shows the output characteristics of the device. Evidently, the device has good FET behavior. Also, the average carrier mobility is 4.8 cm^2^ V^−1^ s^−1^, and the highest value reaches 13.0 cm^2^ V^−1^ s^−1^ (Figure 15c). Peng et al. further quantified the key parameters to observe the effect of molecular crystals to better understand the crystal mechanism.^[^ ^183^ ^]^ First, a quasi‐balanced low‐speed solution was used to shear deposit C_10_‐DNTT. Second, the optimal shear rate for crystal growth based on mass conservation was calculated (Equation 1), and 30 devices were selected at different temperatures and shear rates for test verification. Furthermore, the mean and standard deviation of the saturation mobility were calculated and fitted (Figure 15d). The fitting curve shows a large gap in the saturation mobility at different temperatures and shear speeds. Among them, the highest mobility and the best crystal morphology can be obtained by setting the temperature at 95 °C and the shear rate at 30 µm s^−1^. On this basis, the *I*–*V* curve is fitted to its output and input (Figure 15e,f). Its saturation mobility can reach 12.5 cm^2^ V^−1^ s^−1^, and the switching ratio is 2 × 10^9^. Some key parameters were simulated, and Equation 1 was used as follows:^[^ ^183^ ^]^

(1) $$v_{0}tl\rho =\frac{c}{\rho_{0}}M_{0}\int \int J$$

where *ρ* is the crystal density of C_10_‐DNTT, *t* is the thickness of the organic semiconductor crystals (32 nm) and l is the unit length.

The Navier‐Stokes equation is given as follows:

(2) $$\rho \left(\frac{\partial v}{\partial t}+v\cdot \nabla v\right)=\nabla \cdot \sigma +F$$

The heat transfer equation is given as follows:

(3) $$\rho C_{\rho }v\cdot \nabla T=k\nabla^{2}T$$

The diffusion equation is given as follows:

(4) $$D\nabla^{2}c=0$$

Shi et al. used the gravity‐assisted 2D space constraint method to prepare high‐quality molecular crystals.^[^ ^184^ ^]^ The electrical characteristic curve is shown in Figure 15g,h. Evidently, its saturation mobility reached 0.51 cm^2^ V^−1^ s^−1^, proving that it has a lower contact resistance, which can also be seen in Figure 15k. Figure 15i shows that when *V*_g_ > *V*_th_, electron mobility changes insignificantly. Also, the electron mobility slightly affects *V*_g_, indicating that the device performance is less affected by the contact resistance and interface traps. The channel length was further measured, and the relationship between the electron mobility and the leakage current is shown in Figure 15j. As the channel length increases, no obvious change in electron mobility occurs, and the leakage current changes linearly, indicating that the molecular crystal has a higher quality and the channel resistance decreases uniformly. Figure 15l shows that the mobility decreases with increasing temperature, but between 200 and 300 K, BCB‐SiO_2_ substrates have obvious band‐like transport characteristics, while no banding is observed for the silicon dioxide substrate. This research forms the foundation for realizing high‐performance crystal, gate/optically tuned lateral organic p–n diode. Equation (5) is used to calculate and fit the saturation.^[^ ^184^ ^]^

(5) $$\mu =\frac{2L}{WC_{i}}{\left(\frac{\partial \sqrt{I_{ds}}}{\partial V_{g}}\right)}^{2}\;\mathrm{or}\;\mu =\frac{L}{WC_{i}V_{\mathrm{ds}}}\frac{\partial I_{ds}}{\partial V_{g}}$$

#### 4.1.6 Others

2D CMPs are considerably researched owing to their unique structure and electronic properties. Wen et al. systematically studied the electronic properties of 2D *π*‐CMP materials.^[^ ^185^ ^]^ Wen et al., in their study, showed that 2D *π*‐CMP is more delocalized than 1D materials. Besides, the structural reorganization of the hole and electron transport is very close in a highly delocalized system, and the charge polarization effect exceeds that of 1D materials. These conclusions indicate that this material has good prospects in semiconductors and non‐linear optics. Zhang et al., in their study, showed that CMP (as an advanced porous material) has the advantages of greater substitution, high stability and scalability, and has several applications.^[^ ^186^ ^]^ Additionally, Chen et al. showed that alternative strategies can improve the conductivity and electrochemical stability of the framework structure, and provide multiple electrochemical active sites, providing insights into the energy field.^[^ ^187^ ^]^

### 4.2 Optical Properties

The optical performance of a material refers to its response to electromagnetic radiation and visible light, which is mainly measured by the material's absorption, reflection and transmission characteristics of electromagnetic waves.^[^ ^188^ ^]^ Because of the various complex conditions of energy exchange between electrons and photons, different materials have completely different optical properties.

#### 4.2.1 MOFs

**Figure 16:**
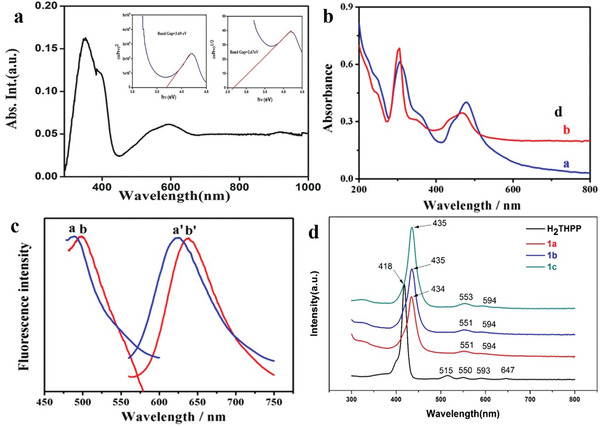
a) Absorption spectrum of materials made using Tauc's plots (inset). Reproduced with permission.^[^ ^189^ ^]^ Copyright 2015, Royal Society of Chemistry. b) UV–Vis absorption spectrum of a) Ru‐MOFNSs and b) Ru(dcbpy)_3_^2+^. c) Fluorescence incentive and shot spectra of a,aʹ) Ru(dcbpy)_3_^2+^ and b,bʹ) Ru‐MOFNSs. Reproduced with permission.^[^ ^191^ ^]^ Copyright 2019, John Wiley. (d) UV–Vis absorption spectra for ligand and 1a–c. Reproduced with permission.^[^ ^192^ ^]^ Copyright 2019, Royal Society of Chemistry.

2D MOFs can absorb a range of photons and generate a photocurrent when revealed to visible light. Roy et al. synthesized a novel thiocyanate bridging 2D MOF, and performed a spectral analysis.^[^ ^189^ ^]^ **Figure** **16**a reveals the assimilation spectra of the composition, which shows the absorption of energy in the UV region of ≈348 nm. The fundamental absorption is very similar to the excitation of electrons from the valence band to the conduction band, so the optical bandgap for the film can also be calculated practically.^[^ ^181^ ^]^ The film bandgap can be calculated as follows:^[^ ^189^, ^190^ ^]^

(6) $$\alpha h\gamma =A\cdot {\left(h\gamma -E_{g}\right)}^{n}$$

where *α* represents the absorption rate, *E*_g_ represents the bandgap, *h* represents Planck's constant, *n* represents the nature of conversion dependent constant and A represents a capacity‐irrelevant number. When *n* = 2 and 0.5, the bandgap numbers are 3.69 and 2.67 eV, respectively. This configuration can act as an “active” material and conduces to exciton movement by charge detachment. The material absorbs photons at 345–390 nm and produces a light current when exposed to visible light.^[^ ^189^ ^]^ Yan et al. prepared 2D MOFs nanosheets (Ru‐MOFNSs) using Zn^2+^ and Ru(dcbpy)_3_^2+^. Ru(dcbpy)_3_^2+^ has an electrochemical luminescence function. The curve in Figure 16b shows the absorption spectrum of light with different wavelengths. Ru‐MOFNSs shows three peaks. The peak positions are at 305, 342–367 and 478 nm, respectively. Curve b in Figure 16b shows the light absorption spectra at different wavelengths of the Ru(dcbpy)_3_^2+^ ligand. Compared with the two curves, the absorption peak of curve a is slightly shifted, indicating coordination between Ru(dcbpy)_3_^2+^ and the carboxyl convergence of Zn^2+^. The fluorescence spectrum is shown in Figure 16c. Evidently, the maximum excitation wavelength and emission wavelength of Ru‐MOFNSs are all shifted to the right compared with pure Ru(dcbpy)_3_^2+^, indicating that some photons have energy transfer.^[^ ^191^ ^]^ Niu et al. used 2D Co‐based MOF with H_2_THPP ligands and porphyrin skeletons and poly‐vinylpyrrolidone (PVP) to form crystalline slices (1a), flower‐like clusters (1b) and ultrathin membranes (1c). Figure 16d shows the ultraviolet–visible (UV–Vis) spectra of 1a, 1b, 1c and free H_2_THPP ligands. At 435 nm, there is a strong sorbet band of the porphyrin skeleton, which is red‐shifted compared to the free H_2_THPP ligand. Owing to the higher D_4h_ symmetry after metallization, there are two collapses at 553 and 594 nm in 1a–c, and the light absorption characteristics of these four substances at 532 nm are negligible.^[^ ^192^ ^]^

#### 4.2.2 COFs

COF‐5 with type II heterojunction arrangement shows obvious valence and lead band shifts, indicating that the space carrier electrons and cavitation agents are effectively separated.^[^ ^193^ ^]^ In TP‐COF, the conduction band shift is still obvious due to the effective confinement of photoexcited electrons. The valence deviation is close to zero owing to the dispersion of the optical excitation holes in the entire structure. Zhou et al. proposed an type‐I heterogeneous junction alignment method in which the side‐wave function is located in the same region, thereby enabling effective spatial carrier aggregation.^[^ ^194^ ^]^ Additionally, because the calculated light absorption peaks of the frameworks are the same as the experimental measurement results, TP‐COF and NiPc‐PBBA‐COF provide reference schemes for the experimental observation of the transmission spectra of these frameworks.^[^ ^194^ ^]^

**Figure 17:**
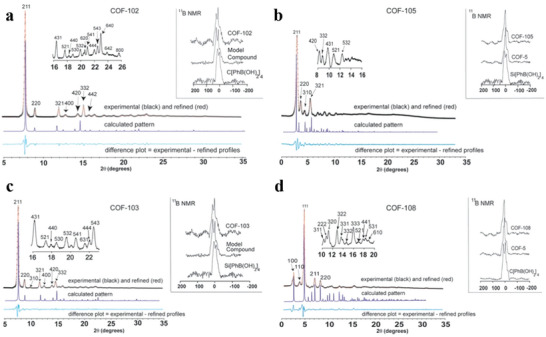
PXRD‐observed and ‐refined contours of a) COF‐102, b) COF‐103, c) COF‐105 and d) COF‐108 samples, which are black and red, respectively. The calculation mode is shown in blue, the difference graph is turquoise (observed minus the simplified outline), and 2*θ* represents the Bragg angle in degrees. The left and right graphs are the observed PXRD spectra, and the B MQ MAS NMR spectra (parts per million) were used to construct the corresponding COF. The middle and bottom lines are the model compound and boric acid, respectively. Reproduced with permission.^[^ ^45^ ^]^ Copyright 2007, American Association for the Advancement of Science.

El‐Kaderi et al. studied these materials through powder X‐ray diffraction (PXRD), elemental microanalysis, microscopy and gas adsorption.^[^ ^45^ ^]^ Extra study remains to verify whether the synthesized product is covalently combined with the designed structure. Comparing the observed synthesis product with it (Figure 17a–d), the results show that the COF sample modeled by PXRD is the expected COF of the ctn or bor (Figure 17d,e) type. There is a clear correspondence between the intensity and position of the peaks observed in the report. The observed PXRD pattern reveals that the narrow line width and low signal‐to‐noise ratio indicate that COF has high crystallinity. It proves that the positions and atomic composition of H, B, C and O in each modeled unit cell were correct.^[^ ^45^ ^]^

#### 4.2.3 HOFs

To obtain 2D HOFs with permanent pores, their structural units are usually designed as high‐rigidity molecules with large *π*‐conjugated aromatic moieties. These molecules are usually excellent fluorescent and phosphorescent dyes,^[^ ^195^, ^196^ ^]^ highlighting that HOF is a promising luminescent material. According to the *π* conjugation, the emission wavelengths of these organic molecules can be distributed in a wide range, which is beneficial to the reasonable design of color‐tunable HOF luminescent materials. Additionally, the high crystallinity of 2D HOFs indicates that organic chromophores are highly ordered.^[^ ^197^ ^]^ Compared with the solution state, the emission behavior of HOFs differs, such as aggregation‐induced emission (AIE).^[^ ^198^ ^]^ In the crystal state, the chromophore is constrained by the close contact between the lattice and some molecules, leading to a spectral shift, loss of vibration structure, expansion of emission range and extension of emission life.^[^ ^199^ ^]^ Additionally, the widely involved intermolecular interactions include *π*–*π*–*π* interactions that cause electronic interactions (such as charge transfer) between phosphors to change the luminescence characteristics.^[^ ^200^ ^]^ Therefore, 2D HOFs show great potential in optical applications.

#### 4.2.4 Perovskite

**Figure 18:**
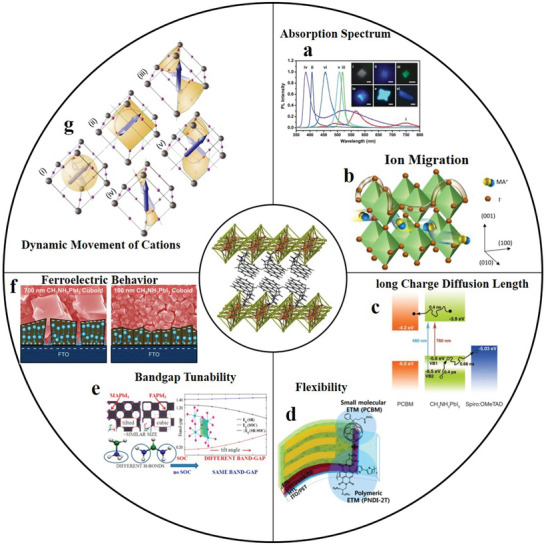
Properties of 2D organic perovskites: a) Absorption spectrum. Reproduced with permission.^[^ ^202^ ^]^ Copyright 2015, American Association for the Advancement of Science. b) Ion migration. Reproduced with permission.^[^ ^180^ ^]^ Copyright 2016, American Chemical Society. c) Long‐charge diffusion length. Reproduced with permission.^[^ ^203^ ^]^ Copyright 2013, American Association for the Advancement of Science. d) Flexibility. Reproduced with permission.^[^ ^140^ ^]^ Copyright 2017, Royal Society of Chemistry. e) Bandgap tunability. Reproduced with permission.^[^ ^179^ ^]^ Copyright 2014, American Chemical Society. f) Ferroelectric behavior. Reproduced with permission.^[^ ^204^ ^]^ Copyright 2015, American Chemical Society. g) Dynamic movement of cations. Reproduced with permission^[^ ^205^ ^]^ Copyright 2015, Nature Publishing Group.

Optical properties occupy an important position in 2D organic perovskite materials. Notably, different optical properties are clearly observed in MAPbI_3_ and FAPbI_3_ perovskites, which is due to the structural variation induced by cations in the material, thus improving the charge transport and red‐shift absorption structure of FAPbI_3_. Ren et al. studied the structure of organic–inorganic perovskites under pressure regulation.^[^ ^201^ ^]^ Under high‐pressure treatment, the bandgap of 2D organic–inorganic halide perovskites can be manipulated. It can be found that the bandgap of (PEA)_2_PbBr_4_ can be significantly changed by pressure‐induced lattice disorder. Also, it is also pointed out that the phase transformation is reversible below 25 GPa, and the resulting lattice distortion returns to its original state after the pressure is released. However, the crystal lattice of the material remains structurally disordered under high pressure of 25 GPa. Therefore, the bandgap of the (PEA)_2_PbBr_4_ can be adjusted from 2.98 to 3.46 eV. The optical properties of 2D organic perovskites can be easily adjusted by controlling the composition, which can open up a new platform to meet the complexity and freedom required by optoelectronic devices. Besides, they are expected to become the choice material for next‐generation artificial neural networks. **Figure** **18** shows some important properties of 2D organic perovskites.

#### 4.2.5 Molecular Crystals

**Figure 19:**
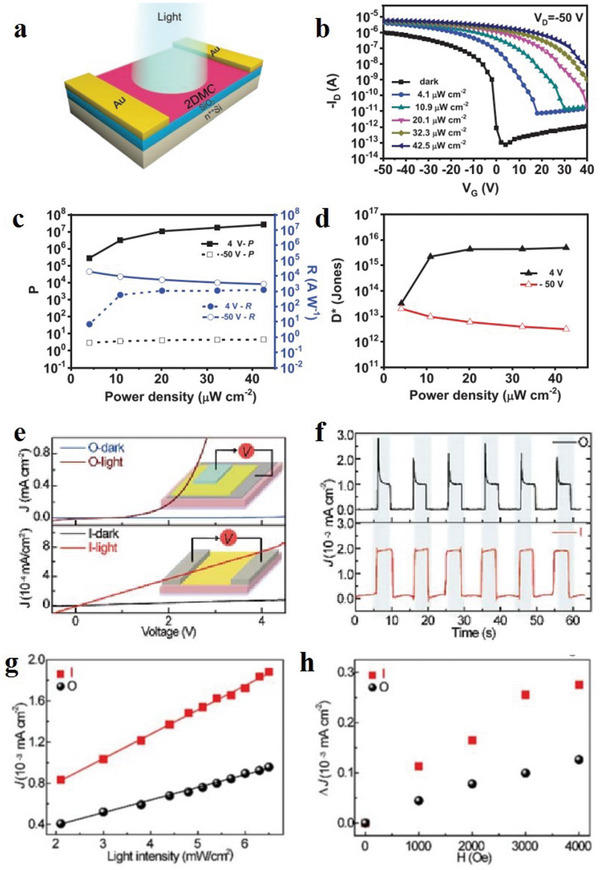
Optoelectronic properties. a–d) The organic phototransistors with bilayer 2DMCs a and e–h) ETC_60_ nanosheet. a–d) Reproduced with permission.^[^ ^206^ ^]^ Copyright 2019, Wiley‐VCH. e–h) Reproduced with permission.^[^ ^208^ ^]^ Copyright 2019, IOP Publishing Ltd.

Molecular crystals are important materials for photodetectors owing to their super‐light sensitivity. Fu et al. proposed a “phase separation” molecular design method to achieve millimeter‐level 2D molecular crystals.^[^ ^206^ ^]^ **Figure** **19**a shows the structure of an organic optoelectronic device with two‐layer 2D molecular crystals. Light tests were performed on the device with different intensities. The results show that as the light intensity increases, the leakage current and threshold voltage also increase (Figure 19b). The photosensitivity (*P* = (*I*_light_ − *I*_dark_)/*I*_dark_) and responsivity (*R* = (*I*_light_ − *I*_dark_)/(*W*_inc_ × *S*)) of the device can be obtained by calculation (Figure 19c) (*W*_inc_ represents the incident light intensity, *S* is the area light area of the channel). When *V*_G_ = 4 V, its photosensitivity can reach 2.58 × 10^7^. When *V*_G_ = −50 V, the responsivity can reach 1.91 × 10^4^ A W^−1^, indicating that the 2D molecular crystal effectively promotes the transfer of charges, with low contact resistance and high charge injection rate.^[^ ^207^ ^]^ Figure 19d uses specific detectivity (D*) as a function of the light density. It can be concluded that when *V*_G_ = 4 V, the illumination intensity = 42.5 µW cm^−2^, and the detection rate culminates to 4.93 × 10^15^ Jones. From the above data, it can be found that molecular crystals have excellent optical properties such as high photosensitivity and high detection rate. Xu et al. prepared the Lvalene‐C_60_ crystal using the improved Langmuir–Blodget technology.^[^ ^208^ ^]^ The molecular crystal has excellent ultraviolet light sensitivity and photoelectric response (Figure 19e–h). Figure 19e shows the *I*–*V* curves of dark and ultraviolet light irradiations with a wavelength of 365 nm. The nanosheets change more significantly under dark and ultraviolet light irradiations, indicating the excellent photosensitivity of the crystal. Through the photocopying of the 2D molecular thin‐film in‐plane and out‐plane orientations shown in Figure 19f under ultraviolet switching conditions, it can be found that its light response significantly exceeds that of the bulk crystal. Moreover, the charge trap density of molecular crystals is very low, and the modulated optical response can be performed by an external magnetic field.

### 4.3 Magnetic Properties

Magnetism is a basic property of matter, which mainly refers to the ability of a material to be attracted by an external magnetic field. The magnetoelectric properties of molecular crystals are discussed below.

#### 4.3.1 MOFs

**Figure 20:**
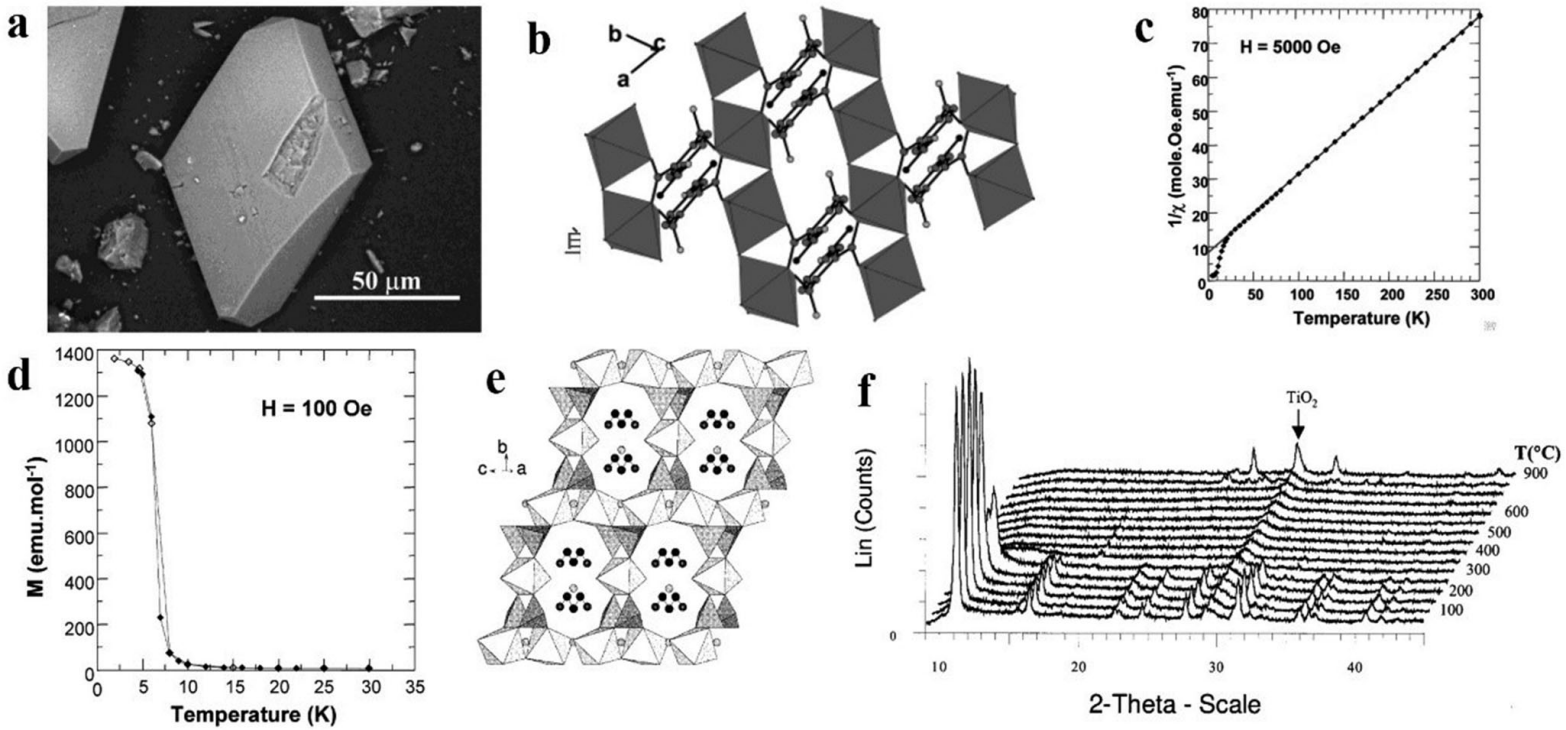
a) SEM image of MIL‐67. b) Chains targeted down the orientation within a layer.^[^ ^116^ ^]^ c) *χ* ^−1^(*T*) bight under a domain of *H* = 0.5 T (the unbent line is relative to the CurieWeiss law). d) M(T) bight under a domain of *H* = 0.01 T, revealing the growth below 8 K. Reproduced with permission.^[^ ^103^ ^]^ Copyright 2003, American Chemical Society. e) Framework of MIL‐283 down the axis.^[^ ^107^ ^]^ f) Thermal X‐ray diffractometry of MIL‐283. Reproduced with permission.^[^ ^104^ ^]^ Copyright 2002, American Chemical Society.

The magnetic properties of MOFs are of interest to researchers. **Figure** **20**a shows the SEM image of MIL‐67. The MIL‐67 crystallizes as a colorless diamond crystal. There are two different octahedral structures of Fe^2+^ in MIL‐67: [FeO_4_(H_2_O)_2_] and [FeO_2_(H_2_O)_4_]. These different octahedral structures form long chains by having a common end of water molecules. These chains are linked by some C_9_O_6_H_4_^2−^ ions to form an organic–inorganic hybrid layer (Figure 20b). Figure 20c shows the magnetic characteristics of MIL‐67. Evidently, MIL‐67 obeys the Curie–Weiss law of paramagnetism at temperatures exceeding 100 K and *χ*_mol_^−1^ = 0.232*T* + 8.38. Below 8 K, MIL‐67 exhibits antiferromagnetic behavior (Figure 20d).^[^ ^103^ ^]^ MIL‐28_3_ is a kind of 2D material (Figure 20e); its inorganic layer comprises TiO_4_(OH)_2_ octahedron, HPO_4_ tetrahedron, PO_4_ and TiO_6_. The units in the structure are connected by Ti(1)O_4_(OH)_2_ octahedrons to form 10 ring perforation tiers. Serre et al. studied the thermal behavior of MIL‐28_3_ using thermal X‐ray diffractometry, and the results are presented in Figure 20f. Below 250 °C, the water does not lead to an effective variation in the characteristics of MIL‐28_3_. However, above 250 °C, the organic matter is destroyed, and the structure of MIL‐28_3_ is destroyed. Amorphous solids are formed above 350 °C. These results indicate that MIL‐28_3_ has excellent structural characteristics.^[^ ^104^ ^]^

#### 4.3.2 Molecular Crystals

**Figure 21:**
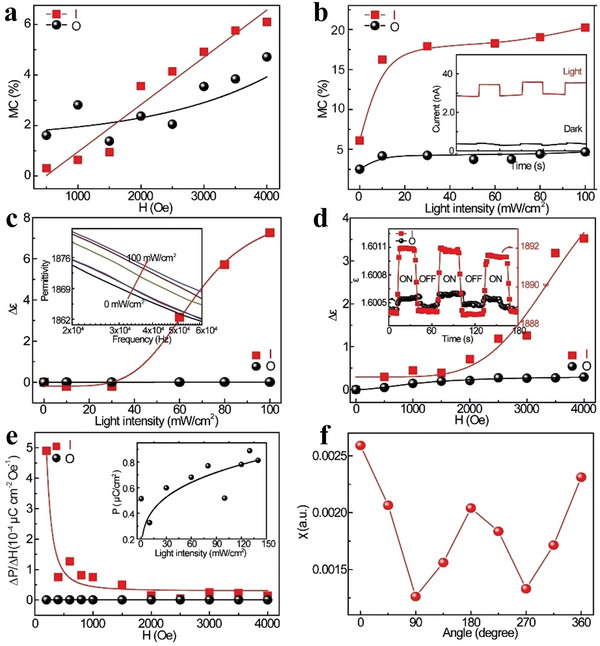
a–f) Anisotropic magnetic field effect, magneto‐electric coupling effect, polarization and magnetization. Reproduced with permission.^[^ ^208^ ^]^ Copyright 2019, IOP Publishing Ltd.

In addition to their electrical and photoelectric properties, independent molecular crystals also have excellent magneto‐electric properties. Xu et al. prepared Lvalene‐C_60_ crystals with superior magnetic properties.^[^ ^208^ ^]^ An in‐depth study of the anisotropic magneto‐electric effect of 2D molecular crystals (**Figure** **21**a,b) reveals that with the negative permeability effect, the mobility of carriers decreases, light transfers molecular charges and the density of triplet excitons and polarons increases, whereas high‐density carriers oriented along the plane have more molecular charge transfer. Figure 21c,d shows the changes in the relative dielectric constants of light intensity and magnetic field, respectively. As the magnetic field and light are enhanced, the magnetic dielectric effect and the photoelectric dielectric constant increase. Besides, the dielectric constant of the in‐plane orientation changes more than that of out‐of‐plane. Also, Figure 21e shows that the dipole exhibits a magneto‐electric coupling effect. When the magnetic field intensity is 200 Oe, the magneto‐electric coupling coefficient oriented in the plane direction culminates to 4.93 × 10^−4^ µC cm^−2^ Oe^−1^. Conversely, there is limited magnetoelectric coupling along the out‐of‐plane orientation. Compared with the out‐of‐plane orientation, the in‐plane orientation has higher magnetization and susceptibility (Figure 20f).

### 4.4 Chemical Properties

The chemical properties of a material are inherent properties that comprise its activity and ability to participate in chemical reactions. Common chemical properties include catalytic, ion exchange and adsorption properties. Because the crystal grains of nanomaterials are tiny, the atomic fraction of the disordered arrangement on the surface of the crystal grains exceeds the percentage of atoms on the surface of the crystalline material, resulting in nanomaterials with excellent chemical properties. The chemical properties of some 2D materials are discussed in detail below

#### 4.4.1 MOFs

2D MOFs are characterized by their large specific surface area, ultrathin thickness and huge exposed metal potential, which makes 2D‐MOF have excellent chemical catalytic properties. The nanoscale thickness contributes to the diffusion of reactants and products. These naked metal sites allow 2D MOFs to act as catalysts for energy transformation processes, such as REDOX and CO_2_ reduction reactions. Kornienko et al. used cobalt porphyrin‐based MOF films as catalysts to reduce CO_2_ to CO, and found that such MOF films have high selectivity and stability.^[^ ^209^ ^]^ Compared with bulk MOFs with stacked structures, 2D MOF nanoflakes have higher catalytic yield, which is a more obvious advantage of 2D MOF. 2D MOFs are characterized by high activity due to their nanometer thicknesses.

#### 4.4.2 COFs

**Figure 22:**
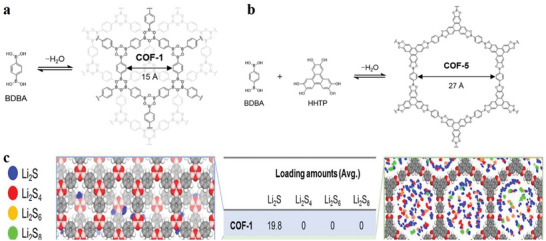
a,b) Chemical structures of a) COF‐1 and b) COF‐5. c) Final binding configuration of Li_2_S*_x_* species (*x* = 1, 4, 6 and 8) in COF‐1 (blue box, left) and COF‐5 (green box, right). Reproduced with permission.^[^ ^210^ ^]^ Copyright 2016, American Chemical Society.

Owing to the chemistry and structural uniqueness, the COF intermediate layer may effectively capture polysulfides without damaging ions. This property ultimately helps to achieve excellent cycle performance. Yoo et al. prepared two COF maps with different pore sizes (**Figure** **22**a,b) as model systems.^[^ ^210^ ^]^ Figure 22c shows the configuration of Li_2_S*_x_* in COF‐1 and COF‐5. The positions of the adsorption points in COF‐5 are the same without any higher priority intermolecular interactions. The results show that within the COF‐5 body, Li_2_S*_x_* molecules are randomly scattered (Figure 22c, right). However, COF‐1 has a small pore size, so only Li_2_S passes through (Figure 22c, left), which means that the pore size can selectively adsorb Li_2_S in COF‐1. Additionally, Yoo et al. also studied the adsorption energy of Li_2_S*_x_* on COF using density functional theory to further clarify the binding behavior of Li_2_S*_x_*.^[^ ^211^ ^]^ The total adsorption energy comprises mainly two aspects: first, the reconstruction of the main structures of COF‐1 and COF‐5 and the structural changes of the Li_2_S*_x_* guest, and second, the bond formation. 2D COFs have very promising applications in many fields, such as gas storage, separation and catalysis.

#### 4.4.3 HOFs

**Figure 23:**
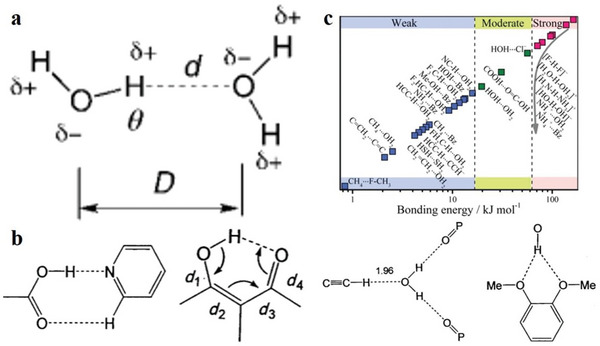
a) Prototype of hydrogen bond: water dimer. b) Different types of hydrogen bonds. Reproduced with permission.^[^ ^212^ ^]^ Copyright 2019, Royal Society of Chemistry. c) Statistics of different types of hydrogen bonds. Reproduced with permission.^[^ ^175^ ^]^ Copyright 2019, Royal Society of Chemistry.

The hydrogen bond is a unique bonding form, usually called a weak hydrogen bond, which is the bond between two structural units X and A when X‐H···A interacts.^[^ ^60^ ^]^ **Figure** **23**a shows the prototype of hydrogen bonds. It is generally believed that chemical bonds should be strong, but hydrogen bonds are not particularly strong because they are generated by the electrostatic action of X‐H···A. Also, because this electrostatic interaction is relatively weak, the hydrogen bond can maintain its unique inherent properties.^[^ ^212^ ^]^ Figure 23b,c shows some common hydrogen bonds and quantitative statistics. If an extreme case is considered, the bond energy of the hydrogen bond ranges from 1–170 kJ mol^−1^, which is < the values of the coordination (90–350 kJ mol^−1^) and covalent (300–600 kJ mol^−1^) bonds. Therefore, compared to coordination and covalent bonds, hydrogen bonds are less rigid and less directional, making the rational design of hydrogen‐bonded organic framework materials more challenging.

#### 4.4.4 Others

2D porous polymers with controllable morphologies present huge challenges in design and synthesis. Recently, scientists have shown that 2D porous polymers have excellent chemical structure and 2D size characteristics. Zhao et al. showed that HCP has large specific surface area and gas storage characteristics, and can be further cross‐linked by optimizing the reaction conditions to realize the separation and storage of gases and molecules.^[^ ^213^ ^]^ Previous studies on CMP mainly focused on chemical strategies and changing the length of organic linkers, and there are few reports on the control of morphology. However, 2D sheet structural polymers have a large surface area and pore size distribution, which are highly advantageous in molecular adsorption and catalysis.^[^ ^214^ ^]^

## 5 Applications of 2D Organic Materials

New 2D organic materials with unique electrical, optical and magnetic properties have potential applications in electrochemistry and micro–nano optoelectronics.^[^ ^215^, ^216^, ^217^, ^218^ ^]^ Also, the integration of silicon‐based semiconductor technology can promote the development of chip technology. The continuous expansion of the family of 2D organic materials provides a foundation for broader research activities and applications and is expected to lead the industrial transformation based on material innovation. Next, the applications of the five materials in different fields are summarized below.

### 5.1 Electronic Application

In recent years, the electronics industry has undergone tremendous changes and made considerable progress. Electronic products have become an indispensable part of people's lives. Therefore, electronic products have developed rapidly in the semiconductor industry and occupy many market resources.^[^ ^219^, ^220^, ^221^ ^]^ Batteries are a research hotspot; thus, relevant reports are constantly updated, and researchers continue to improve the storage capacity and durability of batteries. Additionally, there are related reports on electrochemical sensors, artificial synaptic devices and storage.

#### 5.1.1 Battery

##### MOFs

**Figure 24:**
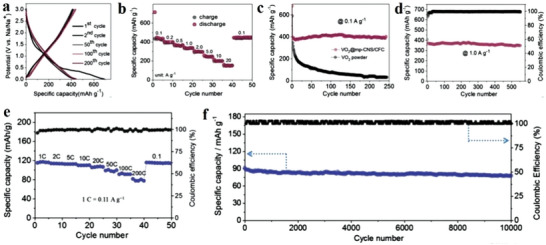
a) Charging and discharging bights. b) Rated property at several current densities. c) Cycle properties of VO_2_@mp‐CNSs/CFC and pure VO_2_ at 0.1 A g^−1^. d) Cycle property of VO_2_@mp‐CNSs/CFC terminal post. e) Rate property of NVP@ mp‐CNSs. f) Cycle property at 100 C of NVP@ mp‐CNSs. Reproduced with permission.^[^ ^222^ ^]^ Copyright 2018, Wiley‐VCH.

2D MOFs can be applied to batteries, and using 2D MOF‐based electrodes can improve the battery performance. Xu et al. reported Na‐ion capacitors (NICs) comprising 2D MOFs. The main method entails converting the MOF array on the surface of the elastic carbon fiber cloth (CFC) into N‐doped mesoporous carbon nanosheets (mp‐CNSs). They used wrapped VO_2_ and Na_3_V_2_(PO_4_)_3_ (NVP) as the anode and cathode electroactive materials on the surface of the nanosheet to obtain VO_2_@mp‐CNSs/CFC and NVP@mp‐CNSs/CFC, respectively. This combination provides ultra‐fast discharge capability, good cycle performance, cycle stability and high‐power characteristics. **Figure** **24**a shows the galvanostatic charge–discharge (GCD) curve with a point window of 0.005–3 V (vs Na/Na^+^). These curves show no significant stagnation during charging and discharging.^[^ ^222^ ^]^ The first charging capacity is 443 mAh g^−1^, the discharge capacity is 692 mAh g^−1^ and the coulomb efficiency is 64%. Figure 24b exhibits the fifth cycle capacity of the electrode. When the electricity density transforms from 0.1 to 20 A g^−1^, the terminal post capacity remains excellent. The cathode also shows good long‐term stability, without significant capacity loss after 250 cycles, with an electricity density of 0.1 A g^−1^ (Figure 24c). Even at a current density of 1 A g^−1^, the capacity attenuation after 550 cycles is only 4% (Figure 24d). The cathode can still provide 70% of the theoretical capacity at the ultra‐high rate of 200 C. Moreover, 10^4^ cycles can be completed at 100 C super high speed (Figure 24e,f).^[^ ^223^ ^]^

In the above synthesis of IRMOF, mesopores were introduced into the original IRMOF‐8 via a competitive coordination strategy. This behavior makes the structure of H‐IRMOF‐8 have both micropores and mesopores, which enhances the utilization value of this material. Mesopores increase the specific surface area and porosity of 2D H‐IRMOF‐8 materials, which promotes the diffusion properties of ions in the battery.^[^ ^24^ ^]^

**Figure 25:**
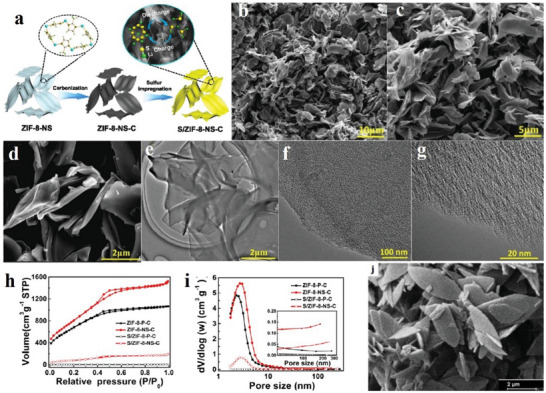
a) Diagrammatical illustration. b–d) SEM and e–g) TEM images of S/ZIF‐8‐NS‐C at disparate enlargement factors. h) Nitrogen adsorption–desorption isotherms. i) Pore diameter distribution curves. Reproduced with permission.^[^ ^28^ ^]^ Copyright 2017, American Chemical Society. j) Leaf‐like morphology observed using FESEM of ZIF‐L. Reproduced with permission.^[^ ^25^ ^]^ Copyright 2018, Elsevier BV.

The coexistence of micropores and macropores is conducive to the motion of Li and the acceleration of electrochemical dynamics of lithium–sulfur batteries in the application of batteries. ZIF‐8‐NS‐C mentioned above has a nitrogen‐rich property that can improve the interaction between the carbon host and lithium–sulfur battery, and the polysulfide can intensify the cycling property of the battery.^[^ ^28^ ^]^ The description of the three products of ZIF‐8‐NS, ZIF‐8‐NS‐C and S/ZIF‐8‐NS‐C are demonstrated in **Figure** **25**a. Figure 25b–d,e–g is the SEM and TEM images of S/ZIF‐8‐NS‐C. No obvious sulfur particle aggregations are observed, indicating that sulfur was dipped into the pores of the carbon carrier. The nitrogen adsorption–desorption isotherms of ZIF‐8‐NS‐C, S/ ZIF‐8‐NS‐C, ZIF‐8‐P‐C and S/ZIF‐8‐P‐C are demonstrated in Figure 25h. The isotherms of ZIF‐8‐NS‐C have a typical mesoporous hysteresis loop, indicating that both micropores and mesopores exist in ZIF‐8‐NS‐C.^[^ ^224^ ^]^ The aperture distribution of ZIF‐8‐NS‐C, S/ ZIF‐8‐NS‐C, ZIF‐8‐P‐C and S/ZIF‐8‐P‐C is revealed in Figure 25i. The maximum aperture size of ZIF‐8‐P‐C is 2.4 nm, and that of ZIF‐8‐NS‐C is 2.8 nm. Additionally, there are large holes of ≈100 nm in ZIF‐8‐NS‐C. The pore structure with the coexistence of micro‐pores and macro‐pores promotes the rapid migration of lithium ions and enhances the power of lithium batteries.^[^ ^28^ ^]^ Figure 25j is the field‐emission SEM (FESEM) diagram of the prepared ZIF‐L. Obviously, the morphology of the ZIF‐L crystal is leaf‐like, with dimensions of 1 µm × 3 µm × 100 nm.^[^ ^25^ ^]^

##### COFs

**Figure 26:**
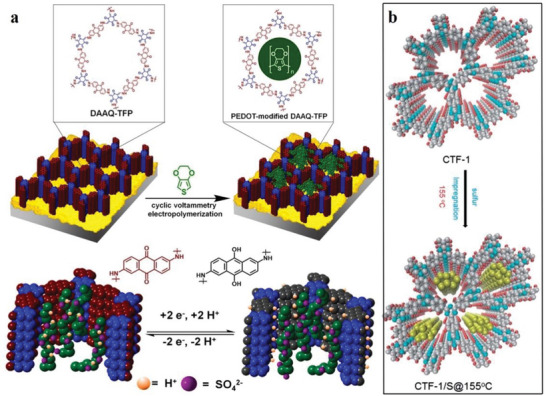
a) Description of pore cross‐section of DAAQ after oxidation and reduction, and preparation of improved COF film. Reproduced with permission.^[^ ^227^ ^]^ Copyright 2016, American Chemical Society. b) Schematic of sulfur immersed in CTF‐1 hole. Reproduced with permission.^[^ ^228^ ^]^ Copyright 2014, Royal Society of Chemistry.

COFs in variable pore environments have semiconductor characteristics, making it possible to have better applications in many fields of energy conversion and energy storage.^[^ ^225^ ^]^ Dichtel et al. reported a COF material condensed by 2,6‐diaminoquinoline and 1,3,5‐tritylthiophene, which exhibits a reversible electrochemical process.^[^ ^226^ ^]^ The synthesized COF‐modified electrode has enhanced capacitance, which does not change much after 5 × 10^3^ charge–discharge cycles. Recently, Mulzer et al. prepared a 2D COF film with redox activity by electropolymerization in the COF hole by 3,4‐ethyl‐dioxoxa acid, giving such agents enhanced conductivity, improved electrochemical response and stable capacitance (**Figure** **26**a).^[^ ^227^ ^]^ Additionally, COFs can also be used as the main material of sulfur impregnation, which positively impacts Li–S batteries. To reflect the good rate and stable cycle performance characteristics, sulfur was introduced into the pores of CTF‐1, and a sulfur electrode was constructed by this method (Figure 26b).^[^ ^228^ ^]^ Sulfur was immersed in the COF and charged and discharged for 200 cycles to enhance the performance of the sulfur cathode.^[^ ^229^ ^]^

##### HOFs

HOFs are excellent substitutes for high‐efficiency proton exchange membrane for fuel cells because of their easy synthesis, processing in solution and structural adjustment, thereby improving the working efficiency of fuel cells.^[^ ^230^, ^231^, ^232^, ^233^, ^234^ ^]^

In 2016, S. K. Ghosh's research group fabricated two cases of HOF materials (HOF‐GS‐10 and HOF‐GS‐11) using aromatic sulfonic acid and guanidine salt.^[^ ^129^ ^]^ This HOF material is ionized by guanidine salt with a sulfonic acid group as a hydrogen bond donor. By using protons as hydrogen‐bond acceptors, aromatic hydrocarbons as pillars and hydrogen bond layers formed by sulfonic acid groups and guanidine salt ions as top plates, 2D framework materials are formed. (Figure 26).^[^ ^129^ ^]^ The authors regard this kind of HOF as a proton source, and HOFs show good proton conductivity (HOF‐GS‐10, *σ* = 0.75 × 10^−2^ S cm^−1^; HOF‐GS‐11, *σ* = 1.8 × 10^−2^ S cm^−1^), which is comparable to commonly used proton exchange membranes (such as Nafion material).

##### CMPs and HCPs

**Figure 27:**
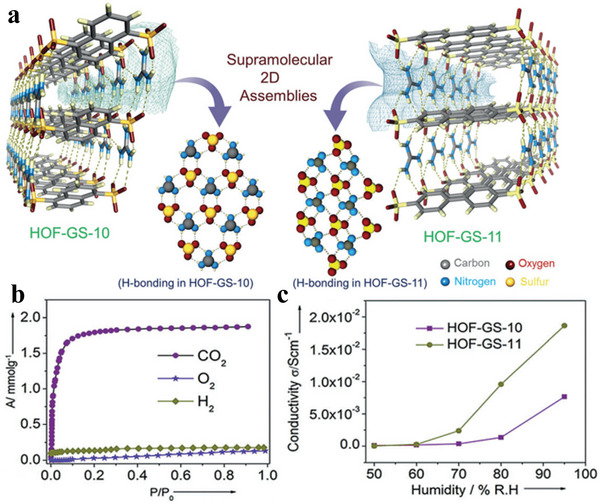
a) Hydrogen‐bonded 2D structure. b) CO_2_, O_2_ and H_2_ adsorption isotherms of HOF‐GS‐10 at 195 K (CO_2_ and O_2_) and 77 K (H_2_). c) Proton conduction of HOF‐GS‐10 and HOF‐GS‐11 at different humidity Reproduced with permission.^[^ ^129^ ^]^ Copyright 2016, Wiley‐VCH.

Because of the severe shortage of traditional energy supply, the world is facing an energy crisis, so more people pay close attention to the research of electrochemical energy storage, especially batteries and supercapacitors, which have been extensively studied.^[^ ^235^, ^236^ ^]^ However, obtaining a storage system with excellent power and capacity performance remains challenging.^[^ ^237^ ^]^ Recent studies have found that mixing batteries and supercapacitors can effectively improve the overall performance of the energy storage system and further provide opportunities for energy storage development.^[^ ^238^ ^]^ Dai et al. prepared electrochromic energy storage materials based on CMP films, and realized a high specific energy storage capacity, reaching 81 mAh g^−1^, which is one of the best results reported for conductive polymers.^[^ ^239^ ^]^ Liao et al. synthesized a CMP network to achieve high‐efficiency supercapacitor energy storage.^[^ ^240^ ^]^ When the power density of the capacitor is 1300 W kg^−1^, the energy density is 60 Wh kg^−1^. Meng et al. reported a structural design strategy based on *π*‐CMP as the anode material of lithium‐ion batteries (LIBs).^[^ ^241^ ^]^ The study shows that the anode material has excellent electrochemical performance, with a discharge capacity of 701 mAh g^−1^, and excellent cycle characteristics. Meng's work provides new insights and directions into the application of 2D CMP materials in LIB technology. Additionally, there are related reports on HCP materials. Chen et al. converted cobalt‐doped graphene HCP into cobalt‐doped porous carbon (Co‐GPC) nanosheets through pyrolysis treatment, and successfully prepared supercapacitors.^[^ ^187^ ^]^ The Co‐GPC exhibited good durability and stability, and has many applications in electrochemical energy storage and conversion (**Figure** **27**).

#### 5.1.2 Electrochemical Sensors

##### MOFs

**Figure 28:**
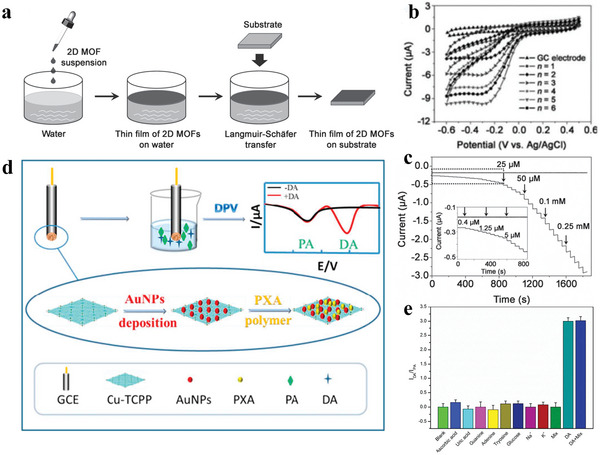
a) MOF film production process. b) Current comparison between bare and Co‐TCPP (Fe) electrodes. c) Current responses of bare and Co‐TCPP (Fe) electrodes. Reproduced with permission.^[^ ^242^ ^]^ Copyright 2016, Wiley‐VCH. d) Sensor preparation process. e) The value of DA/PA in the electrolyte. Reproduced with permission.^[^ ^243^ ^]^ Copyright 2019, Elsevier BV.

The application of 2D MOFs in electrochemical sensors is an important aspect of electrical applications. Several studies have been conducted on the application of 2D MOFs in electrochemical sensors. Zhao et al. applied 2D MOF nanosheets to electrochemical sensors to detect H_2_O_2_ and obtained satisfactory results.^[^ ^242^ ^]^ They assembled 2D M‐TCPP (Fe) (M = Cu, Co and Zn) nanosheets into multiple layer films and changed them to glass–carbon electrodes (**Figure** **28**a). Figure 28b demonstrates the comparison of the influence of the bare and Co‐TCPP (Fe) electrodes on the current in the presence of H_2_O_2_. Co‐TCPP (Fe) nanosheet can quantitatively test H_2_O_2_ with a linear scope of 0.4–50 µm (Figure 28c).^[^ ^87^, ^242^ ^]^ Qiu et al. made a Cu‐MOF‐based 2D MOF nanosheet/Au/polyxanthic acid multiple electrochemical ratiometric sensor for detecting dopamine (DA). Figure 28d presents the schematic of the scheme. First, Cu‐TCPP nanocrystals are scattered on the terminal post, and Au nanoparticles and xanthurenic acid (XA) are assembled on the surface. Cu‐TCPP, AuNPs and XA can produce the electrochemical replies of the DA molecule and sensor. Then, the electrode is inserted into the electrolyte of PA (paracetamol) and DA and applied to the sensing platform by differential pulse voltammetry. Since the effect of PA on the electrochemical signals is negligible, this sensor can be used for DA detection. Figure 28e includes the effect of ascorbic acid, uric acid, guanine, adenine, tyrosine, Na^+^ and K^+^ on the sensor's sensitivity. The results show that these substances or their mixtures do not affect the sensor's sensitivity even at concentrations exceeding 50 times.^[^ ^243^ ^]^

#### 5.1.3 Artificial Synapse Device

##### Perovskite

**Figure 29:**
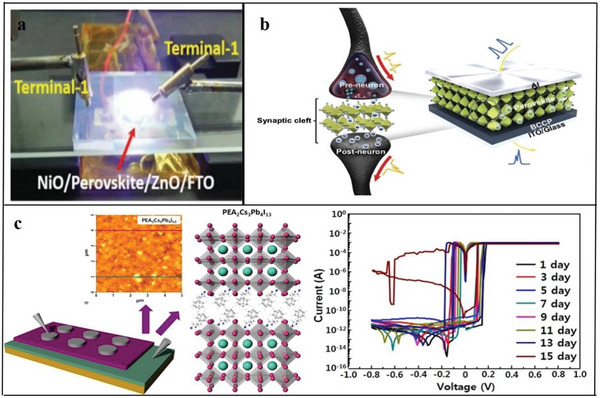
a) Photograph of photo response measurement setup of a two‐terminal device memristor. Reproduced with permission.^[^ ^246^ ^]^ Copyright 2019, Wiley‐VCH. b) Perovskite artificial synapse. Reproduced with permission.^[^ ^244^ ^]^ Copyright 2019, Wiley‐VCH. c) Resistive memory device structure and switching material (PEA)_2_Cs_3_Pb_4_I_13_, and *I*–*V* measurement curve. Reproduced with permission.^[^ ^247^ ^]^ Copyright 2020, Nature Publishing Group.

Because neuromorphic computing operates with low power consumption to stimulate the brain's functions, it has become a core technology that demonstrates future artificial intelligence and overcomes the limitations of traditional structures. To realize the neuromorphic system, various electronic devices must be developed. The emergence of 2D organic perovskite materials provides a good choice for electronic devices. First, 2D organic perovskite materials are used in artificial synaptic devices (**Figure** **29**a). Kim et al. reported similar electronic synaptic devices (Figure 29b).^[^ ^244^ ^]^ Artificial synaptic devices have been prepared using (PEA)_2_MA*_n_*_−1_Pb*_n_* Br_3_ *_n_*_+1_ material, which successfully simulated short‐term plasticity, paired‐pulse facilitation and long‐term plasticity.^[^ ^244^ ^]^ The energy consumption of each synapse calculated by the synaptic device is only 0.7 fJ, which is equivalent to the energy consumption of biological synapses (1–10 fJ per synapse). Sun et al. also demonstrated a new light‐stimulating synapse device based on a 2D lead‐free organic perovskite material.^[^ ^245^ ^]^ The synaptic device showed the photocurrent activation of photostimulation in a neuron‐like manner, and successfully simulated synaptic functions. The materials are expected to become future multifunctional artificial neuromorphic system competitors. 2D organic perovskites are used in memristor devices (Figure 29c). Kumar et al. proposed a novel, highly transparent optical device based on 2D perovskites. The device is turned on or off by a small voltage pulse, and the measured current–voltage has obvious hysteresis, which is in line with the *I*–*V* characteristic of the memristor. This method opens up a new way for fully transparent optoelectronic devices, such as transparent image sensors.^[^ ^246^ ^]^ According to the above report, it can be found that 2D organic perovskites have broad application prospects.

#### 5.1.4 Organic Nonvolatile Ferroelectric Transistor Memories

##### Molecular Crystals

With the development of information technology, ferroelectric memory, especially nonvolatile ferroelectric memory, has gradually come into people's sight, and has been deeply studied. However, the effective acceleration of the charge accumulation and increased carrier mobility in the developmental process remains an important issue. Molecular crystals are important materials to solve this problem.

**Figure 30:**
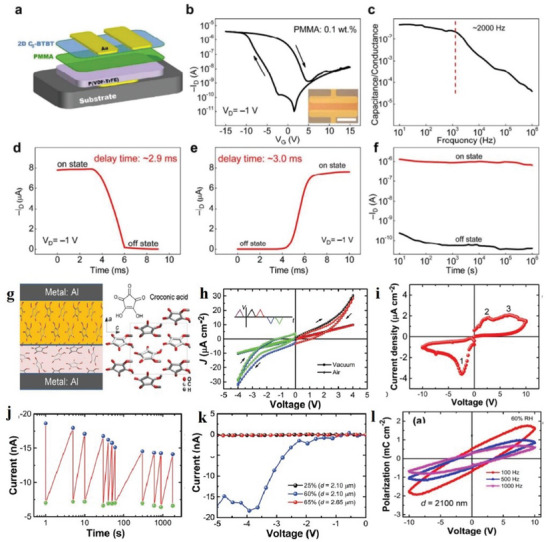
a–l) Application of 2D molecular crystals on organic non‐volatile ferroelectric transistor memories. a–f) Reproduced with permission.^[^ ^248^ ^]^ Copyright 2017, American Chemical Society. g–l) Reproduced with permission.^[^ ^249^ ^]^ Copyright 2018, Wiley‐VCH.

Recently, Song et al. used 2D C_8_‐BTBT molecular crystals as conductive channels based on solutions and ferroelectric materials as dielectrics to form non‐volatile ferroelectric memories.^[^ ^248^ ^]^ **Figure** **30**a shows the schematic of the device structure. Figure 30b–f shows that the device exhibits the current hysteresis characteristics, and its leakage current is as low as 10^−11^ A (Figure 30b), the reading time to the millisecond level is ≈0.5 ms (Figure 30c), the switching time is ≈2.9–3.0 ms (Figure 30d,e), and the retention time is ≈10^6^ s (Figure 30f), indicating that the device runs faster and has excellent nonvolatile performance. In 2018, to prove that ferroelectric polarization can also achieve interface memory switching in uniaxial molecular ferroelectric materials, Hu et al. used croconic acid crystallites to fabricate thin films for capacitive devices.^[^ ^249^ ^]^ It can be found that the device exhibits an obvious ferro‐polarized non‐volatile memory switching behavior (Figure 30i,j). The hysteresis loops are unclamped at a voltage of 0 V, and the hysteresis loops of the first and third quadrants are mostly clockwise, which is a typical ferroelectric behavior. Also, it has a long holding time and can be repeated severally. These features effectively prove that uniaxial molecular crystals can also have stable ferroelectric polarization, thereby preparing non‐volatile ferroelectric memory, which lays the foundation for the preparation of memory devices using uniaxial molecular ferroelectric thin films.

### 5.2 Optical Applications

Optics have developed greatly in applications in the last two decades, such as optical fiber communication, optical disk storage and sensing. Additionally, in electronic computers, photonic technology occupies an important position in external equipment (such as storage, display and output). The application of 2D materials in various fields such as photocatalysis, photosensor and fluorescence emission are summarized below.

#### 5.2.1 Photocatalysis

##### MOFs

**Figure 31:**
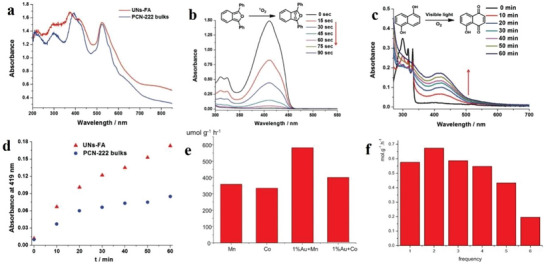
a) UV–Vis divergent reflection spectrum of UNs‐FA and PCN‐222. b) UV–Vis absorption range of 1,3‐diphenylisobenzofuran. c) Photooxidation catalyzed by UNs‐FA. d) Change of A absorption of UNs‐FA and 3D bulks with time. Reproduced with permission.^[^ ^250^ ^]^ Copyright 2018, Wily‐VCH. e) Photocatalytic test photograph of the four materials. f) photocatalytic cycling stability of Au@Mn‐MOF. Reproduced with permission.^[^ ^251^ ^]^ Copyright 2018, Taylor & Francis.

The application of 2D materials in photocatalysis has broad prospects. Severally studies have been conducted on the application of 2D MOFs to photocatalysis. He et al. prepared the ultra‐thin 2D zirconium‐porphyrinic MOF (Zr‐MOF) nanosheet (UNs‐FA) in the presence of formic acid (FA). The UV–Vis absorption spectrum of Zr‐MOF nanosheet and porous coordination network‐222 (PCN‐222) are shown in **Figure** **31**a. This nanosheet can strongly absorb 300–700 nm light waves, indicating that the nanosheet can absorb photons under visible light irradiation. The high light‐trapping capacity of porphyrins is used to form singlet oxygen (^1^O_2_) in photocatalysis. To verify the production of ^1^O_2_, they used 1,3‐diphenylisobenzofuran for experiments. Under the irradiation of light and in the presence of UNs‐FA, the characteristic absorption of the nanosheet decreased, indicating the production of ^1^O_2_ (Figure 31b). UNs‐FA can also be used as a photo‐oxidant of 1,5‐dihydroxynaphthalene catalyst for synthesizing juglone. As shown in Figure 31c, the absorption at 419 nm increases continuously, indicating the formation of juglone. Figure 31d indicates that UNs‐FA has a higher catalytic activity than PCN‐222 block.^[^ ^250^ ^]^ Luo et al. synthesized a 2D Mn‐MOF [Mn_2_(TylP)_4_]*_n_*, Co‐MOF and Au nanoparticle‐coated Mn‐MOF (Au@Mn‐MOF) and Co‐MOF (Au@Co‐MOF), and compared the photocatalytic experiments of hydrogen production between these four materials. The experiment was performed under LED light, [Mn_2_(TylP)_4_]*_n_* was the activator, fluorescein was the photosensitizer and triethyl‐amine solution was the electron donor. With a small amount (2 mg) of [Mn_2_(TylP)_4_]*_n_*, a large amount of hydrogen is obtained within 3 h. Figure 31e indicates that the catalytic capacity of Mn‐MOF is superior to that of Co‐MOF, and the catalytic capacity of Au@Mn‐MOF is superior to that of Au@Co‐MOF. Au@Mn‐MOF also possess good cycling property, with 80% catalytic efficiency after five cycles (Figure 31f).^[^ ^251^ ^]^

##### HCPs

Global CO_2_ emissions have caused a severe greenhouse effect. Therefore, it is necessary to find alternative methods to convert CO_2_ into less or nontoxic substances. Among them, the method of light reduction of CO_2_ into chemical fuel has attracted more attention.^[^ ^252^ ^]^ Presently, articles have reported that heterogeneous systems can reduce CO_2_ with high stability and recyclability, so there is an urgent need to explore high‐performance heterogeneous photocatalysts.^[^ ^253^ ^]^ Wang et al. reported a porous HCP‐TiO_2_‐graphene composite structure, and the results showed that the CO_2_ absorption capacity reached 12.87 wt%, and the CH_4_ generation was 27.62 µmol g^−1^ h^−1^, reaching 83.7%.^[^ ^254^ ^]^ This result realizes solar energy conversion into fuel by combining 2D HCPs and photocatalysts, and expands the application potential of porous materials. Additionally, Choi et al. successfully designed the morphology of HCP using a nonheterogeneous catalytic system and showed good catalytic performance.^[^ ^255^ ^]^

#### 5.2.2 Optical Sensors

##### MOFs

**Figure 32:**
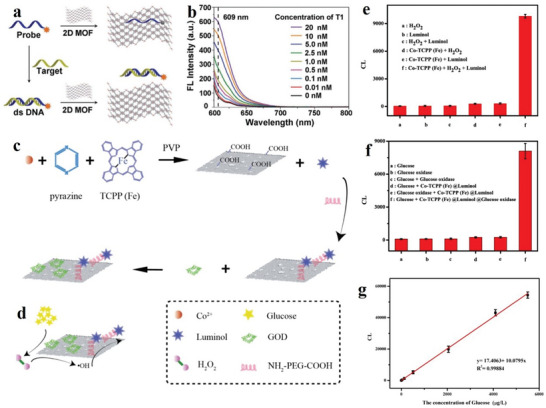
a) Schematic explanation of splendent DNA sensor. b) Scope of DNA investigation with accretion of disparate concentrations of objective DNA (T1). Reproduced with permission.^[^ ^256^ ^]^ Copyright 2015, Wiley‐VCH. c) Course preparation of CL sensor. d) Glucose detection method. (e) Photic properties of Co‐TCPP(Fe)@luminol. f) Photic properties Co‐TCPP(Fe)@luminol@GOD. g) Standard curve of CL and glucose concentration. Error bars manifest the normal difference of three repetitions. Reproduced with permission.^[^ ^257^ ^]^ Copyright 2019, American Chemical Society.

The application of 2D MOFs to optical sensors has been greatly investigated by scholars. Zhao et al. reported a 2D MOF Cu‐TCPP nanosheet fluorescence sensor that could be used as a sensing terrace for Deoxyribonucleic acid (DNA).^[^ ^256^ ^]^ In the non‐entity of objective DNA, single‐stranded DNA (ssDNA) labeled with Texas Red was adsorbed to the surface of the MOF, and the red fluorescence was extinguished owing to the fluorescence resonance energy transfer. When this ssDNA binds to the complementary objective DNA to become double‐stranded DNA (dsDNA), the dsDNA is delivered back onto the nanosheet outside. The double‐stranded structure of dsDNA leads to the weak interaction between DNA and nanocrystals, which can restore fluorescence (**Figure** **32**a). This feature enables the nanometer chip to be applied to the quantitative detection of target DNAs (Figure 32b).^[^ ^256^ ^]^ Zhu et al. synthesized 2D MOF Co‐TCPP (Fe). They labeled the nanochip as Co‐TCPP(Fe)@luminol@God with luminol and glucose oxidase (GOD), and applied it to the chemiluminescence (CL) sensor, which can be used to test the glucose content in urine.^[^ ^257^ ^]^ The schematic of the Co‐TCPP(Fe)@luminol@God CL sensor is shown in Figure 32c. The process of detecting glucose is shown in Figure 32d. 2D MOFs can catalyze the disintegration of H_2_O_2_ and oxidize luminol to become a chemiluminescence signal. Using experiments, they demonstrated that only Co‐TCPP(Fe)@luminol could enhance the electrochemical signal in the presence of H_2_O_2_ (Figure 32e), demonstrating that luminol has been combined to Co‐TCPP(Fe). As shown in Figure 32f, adding a glucose solution to Co‐TCPP(Fe)@luminol@God could enhance the CL signal, indicating that this 2D MOF can be used as a chemiluminescence sensor for glucose detection. To facilitate the comparison, the researchers also presented the standard glucose detection map (Figure 31g).^[^ ^257^ ^]^

#### 5.2.3 Fluorescence Emission

##### HOFs

**Figure 33:**
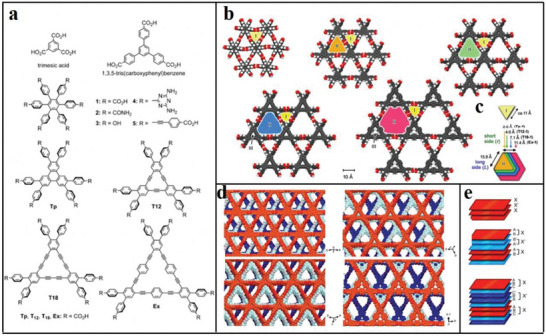
a) Ligand structure. b) Pore diameters of different structures. c) Comparison diagram of pore diameters of different structures. d) Stacking structural diagrams of different structures. e) Schematic representation of three stacking modes in different structures. Reproduced with permission.^[^ ^125^ ^]^ Copyright 2016, American Chemical Society.

**Figure 34:**
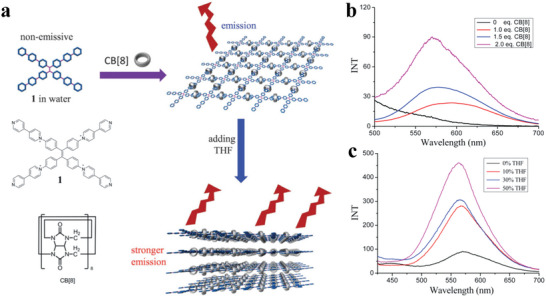
a) Chemical structure of compound 1 and CB and schematic of 2D HOF formation and fluorescence gradual enhancement process. b,c) Fluorescence spectra of different materials. Reproduced with permission.^[^ ^261^ ^]^ Copyright 2015, Royal Society of Chemistry.

HOF is a promising luminescent material. Because of its high rigidity, the large p‐conjugated planar part is widely used to construct permanent porous HOFs, which is also an excellent carrier for high luminescent materials.^[^ ^195^, ^196^ ^]^ Hisaki et al. used various C3‐symmetric polycarboxylic acids to develop robust HOFs with significant porosity and specific surface area characteristics (**Figure** **33**a–e).^[^ ^125^, ^132^, ^258^, ^259^, ^260^ ^]^ Each hexacarboxylic acid is connected with six neighboring ligands through six COOH dimers with multiple O–H–O interactions to form a 2D hexagonal network. Through the interaction of *π*–*π* and C–H···*π*, each H‐bonded layer further overlaps an adjacent layer, thus presenting open frameworks with two different holes, which are triangular and irregular hexagonal shaped. These HOFs have different fluorescence spectra, where the maximum wavelength is 416–546 nm, and the quantum yields reach 5.5–25%. In 2015, Zhao's research group used cucurbit ring and AIE compounds to construct a 2D hydrogen‐bonded organic skeleton material (**Figure** **34**).^[^ ^261^ ^]^ After self‐assembly, the material can emit fluorescence, whereas the original AIE ligand has no fluorescence phenomenon in the monomer. Especially, when tetrahydrofuran is added to the material, the fluorescence phenomenon of the material is obviously enhanced. At first, the AIE ligand and cucurbit ring are self‐assembled to form a monolayer HOF material, which destroys the fluorescence emission of non‐emitting organic‐building blocks. Afterwards, because of the material's 2D layered structure, the ligands were piled up, which further enhanced the fluorescence emission performance of the material.

### 5.3 Optoelectronic Applications

2D organic materials have been deeply researched owing to their atomic‐scale thickness and unique optoelectronic properties. Based on this characteristic, the application field of optoelectronic devices has shown bright prospects. The outstanding representatives of optoelectronic applications are solar cells and LEDs.

#### 5.3.1 Solar Cells

Perovskite: 2D organic perovskite materials are greatly applied in solar cells. There are continuous research and developments devoted to this area. Tan et al. enhanced the efficiency of the device by adjusting the organic spacer cation.^[^ ^262^ ^]^ Here, the combination of F_3_EA^+^ and BA^+^ cations as a mixing spacer promotes the self‐assembly of the 2D perovskite material. The solar cell has a power conversion efficiency (PCE) of 12.51%. Fu et al. studied the effects of PEA, F‐PEA and MeO‐PEA on solar cells.^[^ ^263^ ^]^ Interestingly, the perovskite achieved a lower bandgap and lower binding energy under the influence of F‐PEA or MeO‐PEA. Because of the excellent charge transfer capability and the lowest bandgap, the F‐PEAI‐based perovskites solar cells (PSC) have a PCE of 14.5%, and still have superior stability in a humid environment. The PSC still retains 90% of the original PCE after 40 days. Chen et al. used the steam method to react perovskite with BEAI_2_ materials to obtain a highly efficient and stable PSC.^[^ ^264^ ^]^ The PCE of the perovskite solar cell reached 19.58%. Ye et al. reported a 2D perovskite passivation agent project based on a wide bandgap (≈1.68 eV) perovskite.^[^ ^265^ ^]^ The biggest disadvantage of 2D organic perovskites is the long and bulky organic cations, hindering the transfer of charges. Here, F_5_PEA^+^ was introduced to replace part of PEA^+^. The study results indicated that the perovskite based on the mixed ammonium 2D‐PPA has a five‐fold and three‐fold increase in charge transport on and in the plane, respectively, and the performance was improved from 19.58% to 21.10%. Additionally, the stability was also improved, which is the highest performance of wide bandgap PSC.

#### 5.3.2 LEDs

##### Perovskites

**Figure 35:**
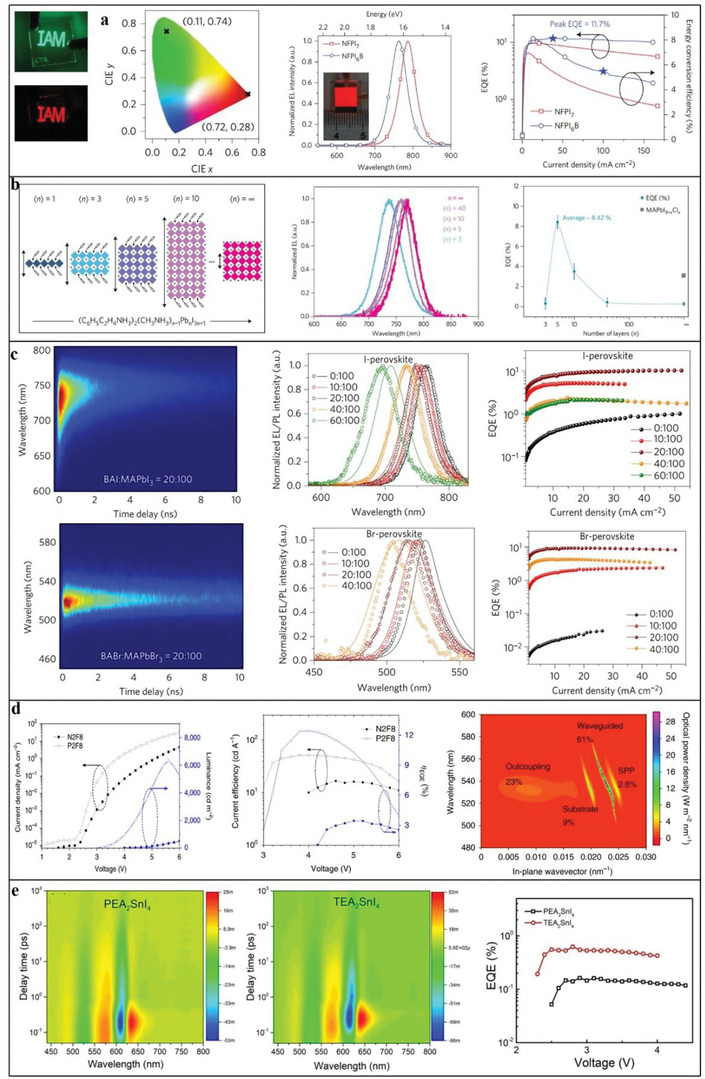
a) Perovskite spectrum, EQE and energy conversion efficiency versus current density. Reproduced with permission.^[^ ^267^ ^]^ Copyright 2016, Nature Publishing Group. b) Unit cell structure and EL spectrum of perovskite with different <*n*> values. Reproduced with permission. Reproduced with permission.^[^ ^268^ ^]^ Copyright 2016, Nature Publishing Group. c) Time‐resolved PL spectra for BAI:MAPbI_3_ (20:100) and BABr:MAPbBr_3_ (20:100), PL/EL spectra and EQE of BAI:MAPbI3 and BABr:MAPbBr3 with different molar ratios. Reproduced with permission.^[^ ^269^ ^]^ Copyright 2017, Nature Publishing Group. d) PeLED current density and EQE. Reproduced with permission.^[^ ^270^ ^]^ Copyright 2020, Nature Publishing Group. e) Fs‐transient absorption spectrum of PEA_2_SnI_4_ and TEA_2_SnI_4_ films excited at 400 nm, and the EQE characteristics of PeLED devices. Reproduced with permission.^[^ ^139^ ^]^ Copyright 2020, American Chemical Society.

Although the traditional LED technology is relatively mature and has high luminous efficiency, it cannot epitaxially grow and prepare large‐area flexible devices. However, LEDs based on 2D organic perovskites can overcome the difficulties of traditional LEDs, and provide LEDs with low cost, large area and high efficiency. The earliest use of 2D organic perovskite materials in LED research was in the mid‐1990s. Era et al. synthesized the first batch of LEDs based on 2D‐RPP (PEA_2_PbI_4_) with high luminosity (measured to the intensity of light emission exceeding 10^4^ cd m^−2^ under liquid nitrogen).^[^ ^266^ ^]^ With the excellent characteristics of perovskite materials, the interest in LED research has intensified. Wang et al. used a solution method to process perovskite materials at low temperatures to obtain direct bandgap semiconductors.^[^ ^267^ ^]^ The prepared thin film with self‐organized multi‐quantum well (MQW) has been successfully applied to perovskite light‐emitting diodes (LEDs). LEDs have good stability and high‐power performance characteristics, as well as an extremely high‐light extraction efficiency (EQE) of 11.7% and energy conversion efficiency of 5.5% (**Figure** **35**a). Because perovskite has excellent mobility and free carriers, it helps non‐radiation centers to effectively capture non‐equilibrium charge carriers. Yuan et al. used several grains with different quantum sizes to obtain perovskite materials, and finally concentrated the light excitation to the luminous body with the lowest bandgap in the mixed perovskite.^[^ ^268^ ^]^ Here, the EQE of LEDs made with new materials is 8.8%, which is by far the brightest and most efficient solution‐processing near‐infrared LED (Figure 35b). Based on the low nonradiative recombination rate and tunable bandgap characteristics of perovskite, Xiao et al. successfully prepared self‐assembled nano‐sized crystallites to achieve high‐efficiency perovskite LEDs.^[^ ^269^ ^]^ The growth of perovskite grains is effectively restricted during the film formation process by adding ammonium halide, thereby forming an efficient luminous body. By calculating the EQE of the perovskite‐type LED, the EQE values of the methyl ammonium lead iodide and bromide systems are 10.4% and 9.3%, respectively, and the life and stability are significantly improved (Figure 35c). Perovskite LEDs have high color purity and high performance, and are expected to be used in next‐generation lighting and displays. Qin  et al. studied a work in which green quasi‐2D chalcogenide devices emitted very critical are triplet‐state excitons.^[^ ^270^ ^]^ The EQE of the obtained green (527 nm) LED was 12.4% and the current efficiency reached 52.1 cd A^−1^ (Figure 35d). One of the biggest difficulties for commercial developers of perovskite materials is the presence of lead. Therefore, finding an element that can replace lead is a direction for future development. Wang et al. reported that two types of 2D tin‐based perovskites are used as pure red electroluminescent devices.^[^ ^139^ ^]^ They found that TEA_2_SnI_4_ greatly improves the performance of LED. The lowest turn‐on voltage of the LED displaying TEA can reach 2.3 V, and the highest bit of EQE is 0.62%, which is by far the highest efficiency and brightness of a pure red LED (Figure 34e).

#### 5.3.3 Photoelectric Detectors

##### Perovskites

2D organic perovskite materials also have abundant applications in photodetectors. Min et al. fabricated flexible 2D‐layered organic halide photodetectors with heterostructures on polyimide substrates.^[^ ^271^ ^]^ The material was developed for inkjet‐printing photodetector equipment. The reported photodetector has high light responsiveness and stability and can be easily converted into a flexible solar cell. Wei et al. designed a promising quasi 2D organic–inorganic hybrid perovskite/IGZO heterostructure flexible phototransistor.^[^ ^272^ ^]^ The device has a wide light response (457–1064 nm), a high response of 3.1 × 10^5^ AW^−1^ and a record detection coefficient of ≈5.1 × 10^16^ Jones under light.

##### Molecular Crystals

In recent years, high‐sensitivity photodetectors have been widely used in military, medical, astronomical, agricultural production and environmental monitoring fields. In optoelectronic devices, organic semiconductors, such as small molecule polymers, are important photosensitive materials. These materials have unique advantages, such as adjustable bandgap, effective field‐effect modulation, high carrier mobility, and low preparation costs. To improve the performance of optoelectronic devices, reduce noise, increase sensitivity and ensure the effective charge injection in the internal charge transport behavior, researchers often use transistors based on high‐quality 2D molecular crystals. 2D molecular crystals have ultra‐thin structural layers, which can achieve full depletion, super light absorption and other effects, hence significantly improving the performance of optoelectronic devices.

**Figure 36:**
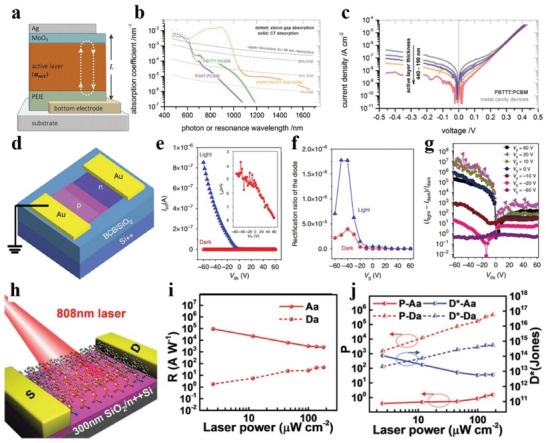
(a)–(c) Application of 2D molecular crystals on optoelectronic detectors. Reproduced with permission.^[^ ^273^ ^]^ Copyright 2017, Wiley‐VCH. (d)–(g) Reproduced with permission.^[^ ^184^ ^]^ Copyright 2018, Nature publishing group. (h)–(j) Reproduced with permission.^[^ ^154^ ^]^ Copyright 2018, Wiley‐VCH.

Recently, Tang et al. used a solution absorbed by charge transfer (CT) to treat bimolecular crystals to fabricate a near‐infrared photodetector.^[^ ^273^ ^]^ The device achieves lower parasitic loss and dark current, which shows that the device has better spectral selectivity and ductility, and the spectral resolution can reach 14 nm (**Figure** **36**a–c). This work shows that organic materials can be photoelectrically detected in the near infrared. Compared to traditional photodetectors, this detection method is simpler, and the device structure is more optimized. In 2018, Shi et al. prepared a large‐area n‐type molecular crystal using a gravity‐assisted 2D space constraint method, and the mobility reached ≈1.24 cm^2^ V^−1^ s^−1^.^[^ ^184^ ^]^ Under dark conditions, the device can achieve rectification ratios of 4×10^5^ and 1.8×10^6^. Under light conditions, the photosensitivity can reach 10^7^ (Figure 36d–g). This research can implement more complex devices, laying the foundation for further research investigation on devices, such as effective rectifiers and photodetectors. Recently, Wang et al. used the simple drip method to prepare novel n‐type organic molecular crystals used in transistors.^[^ ^154^ ^]^ The charge mobility of the device is 1.36 cm^2^ V^−1^ s^−1^, and the photocurrent on‐to‐off ratio and specific detectivity of the phototransistor change with the light intensity change. In the accumulated area, it can get to 9 × 10^4^ and 1.3 × 10^3^ A W^−1^. In the depleted area, the maximum value of *P* and D* can reach 5× 10^5^ and 6 × 10^14^ Jones, which is much better than ordinary phototransistors (Figure 36h–j).

### 5.4 Magnetic Applications

Magnetic sensors are important in many fields, such as science, technology and military affairs. Here, the magnetic applications of molecular crystal materials are summarized.

#### 5.4.1 Magnetic Sensor and Energy Harvester

##### Molecular Crystals

**Figure 37:**
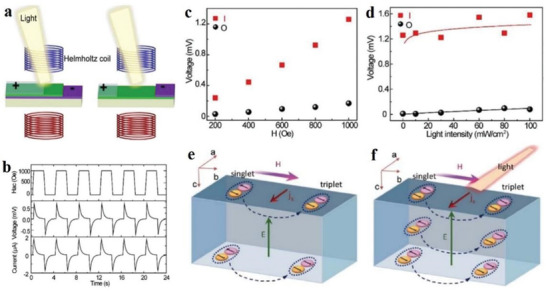
(a)–(f) Magnetic energy harvest and sensing. Reproduced with permission.^[^ ^208^ ^]^ Copyright 2019, IOP Publishing Ltd.

2D molecular crystals with magneto‐electric‐coupling effects are often used to obtain magnetic energy and sensors. Xu et al. prepared an ETC_60_ thin film to observe its magnetic energy collection ability.^[^ ^208^ ^]^ **Figure** **37**a shows two sets of electrodes used to measure the in‐plane and out‐plane through the coil. An AC magnetic field is formed, and the evolution of the relevant magnetic field is shown in Figure 37b. After testing, the device has a higher sensitivity at 200 OE, and with the increase in light intensity and magnetic field strength, the in‐plane orientation is better than the out‐of‐plane magnetic energy acquisition ability (Figure 37c,d). Also, the external magnetic field can induce the spin current (Figure 37e). Besides, the light further stimulates the charge transfer density in the film, thereby increasing the spin current. The magneto‐electric (ME) coupling is greater, and the magnetic energy collection ability is stronger (Figure 37f). Therefore, 2D molecular crystal materials are often used to replace magnetic sensors or energy harvesters.

#### 5.4.2 Organic Spintronic Devices

An approach has been adopted to obtain nonmagnetic covalent organic materials by high‐concentration chemical doping. For this purpose, two things are to be needed, the COFs should have narrow energy band and weak electronic coupling. The dopants orbital energies should be closed to COFs such as p‐dopants of I and Br and COFs, whose valence bands are localized on the site of pyrene node. These nodes not only inject charges but also localize the electronic states and smooth the energy bands, which form a supramolecular charge‐transfer complex. Two new COFs, namely PPy‐CCNaph and PPy‐CC‐4FPh are designed, which possess iodine doping and prospective magnetism. There are also observed anisotropic magnetic interactions in these types of 2D COFs. Where interlayer ferromagnetic interactions are represented through space exchange and intralayer antiferromagnetic interactions are represented by bond exchange. The magnetic coupling and spin channels can be easily modulated by chemical building blocks in both half‐metallic and metallic structures. These results revealed how to develop new spin‐polarized COFs. These findings led to the pathway for nonmagnetic organic materials, which have potential applications in organic spintronic devices.^[^ ^274^ ^]^

#### 5.4.3 2D Organic Materials for Biomedical Applications

A vast number of experimental studies on electronics and spintronic revealed graphene's potential applications in biomedicines. To make graphene hydrophilic, its surface should be functionalized with a suitable functional group. This kind of functionalization enhanced the colloidal stability and is highly required for biomedical applications. GQDs have been recognized as less toxic graphene‐based systems. Furthermore, it is guessed that due to a little different spins nature in sp‐based systems compared to that of the customary d‐block‐element‐ or f‐block‐element‐containing magnetic materials, and spin relaxation may provide interesting contrast properties in T1‐ or T2‐weighted magnetic resonance images.^[^ ^275^ ^]^

### 5.5 Chemical Applications

The chemical applications of 2D materials are very extensive. With the continuous advancement of science and technology and the electronics industry, chemical applications and high‐tech technologies have become increasingly inseparable. Some applications of 2D organic materials in chemistry, including chemical catalysis, chemical adsorption and chemical molecules, are discussed below.

#### 5.5.1 Catalysis Applications

##### MOFs

**Figure 38:**
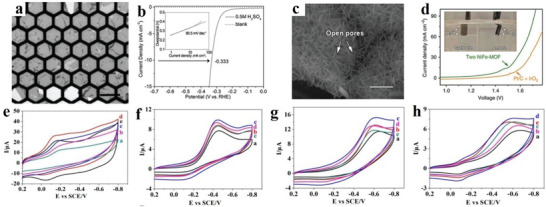
a) TEM icon of unilaminar Ni‐THT MOF. Reproduced with permission.^[^ ^276^ ^]^ Copyright 2015, Wiley‐VCH. b) Comparison of hydrogen evolution reaction curve of Ni‐THT MOF and curve of blank glass–carbon magnetic disc electrode in 0.5 m H_2_SO_4_. The inset reveals that the comparative Tafel slope is 80.5 mV decade^−1^. c) SEM icon of NiFe‐MOF electrodes; plotting scale = 1 mm. Reproduced with permission.^[^ ^277^ ^]^ Copyright 2017, Nature Publishing Group. d) Comparison of NiFe MOF electrode and Pt/C and IrO_2_ electrode. The illustration shows the position of hydrogen and oxygen bubbles on the electrode. *C*–*V* curves of e) Cu‐TCPP nanofilm/GCE and f) Cu‐TCPP nanosheet/GCE. *C*–*V* curves of g) Co‐TCPP nanofilm/GCE and h) Co‐TCPP nanosheet/GCE. Reproduced with permission.^[^ ^23^ ^]^ Copyright 2019, Royal Society of Chemistry.

Herein, we report some catalytic applications of 2D MOF. The thickness of 2D ultrathin Ni‐THT MOF prepared by Feng et al. is only 0.7–0.9 nanometers, and the transverse size is of several square millimeters (**Figure** **38**a).^[^ ^87^ ^]^ The starting and working points of this nanosheet as the catalyst for hydrogen evolution reaction are 110 and 333 mV, respectively, and the current density can reach 10 mA cm^−2^ (Figure 38b).^[^ ^276^ ^]^ These results are better than that of previously reported graphene‐doped catalysts. The NiFe‐MOF array prepared by Zhao et al. also showed excellent catalytic activity.^[^ ^277^ ^]^ The transverse size of the nanosheet array is several hundred nanometers, and the spacing of the nanosheets is about tens of nanometers (Figure 38c). The catalytic activity of the nanosheet array was found in both the hydrogen and oxygen evolution reactions. In the integrated water pyrolysis experiment, the nanosheet was used as both the cathode and anode catalyst. The current density at 1.55 V was 10 mA cm^−2^, which was 70 mV < the reference catalyst comprising Pt–C cathode and IrO_2_ anode (Figure 38d).^[^ ^277^ ^]^

Cu‐TCPP films have important applications in bioenzymes. Figure 38e exhibits the *C*–*V* curve of Cu‐TCPP nanofilm/glassy carbon electrode (GCE) in catalyzing the H_2_O_2_ reduction reaction (it has been proven that GCEs have no catalytic effect; the numerical value of deposition cycles (*n*) = 1, 2, 3, 4 and 5). Evidently, at −0.2 V, a prominent reduction peak is observed, and the reduction peak current is the largest when *n* = 4.^[^ ^23^ ^]^ Figure 38f shows that the Cu‐TCPP nanosheet/GCE catalyzed the H_2_O_2_ reduction reaction on the *C*–*V* curve (GCEs have no catalytic effect; *n* = 1, 2, 3, 4 and 5). The reduction peak of H_2_O_2_ occurs when the point position is −0.45 V, and the maximum reduction peak current occurs when *n* = 4. Similarly, researchers also used 2D MOF Co‐TCPP nanofilms and nanosheets to catalyze the REDOX reaction of H_2_O_2_. Figure 38g,h shows the *C*–*V* curves of 2D MOF Co‐TCPP nanofilm/GCE and Co‐TCPP nanosheet/GCE in catalyzing H_2_O_2_ reduction reactions (GCEs have no catalytic effect; *n* = 1, 2, 3, 4 and 5). For the 2D MOF Co‐TCPP nano‐film/GCE, the reduction peak of H_2_O_2_ occurs when the point position is −0.6 V, and the reduction peak current is the largest when n = 3. For the 2D MOF Co‐TCPP nanosheets/GCE, the reduction peak of H_2_O_2_ occurs when the point position is −0.56 V, and the reduction peak current is the largest when *n* = 4.^[^ ^23^ ^]^

##### COFs

For homogeneous catalysis and heterogeneous materials, COFs are more effective in reducing the distance between them. For example, catalytically active BF‐COF‐1 and BF‐COF‐2 based on alkaline energy COFs are used as catalysts for shrinkage reactions.^[^ ^278^ ^]^ In 2015, Lin et al. prepared a compound comprising a charged niobium oxide catalyst and 1,4′‐phthalaldehyde diphenyl, 4′‐formaldehydes through an amine‐binding reaction.^[^ ^279^ ^]^ This catalytic material performs well in the preparation of CO by the electrochemical reduction of CO_2_ in an aqueous solution.^[^ ^279^ ^]^ The addition of COFs to catalytic species has also attracted significant research attention. Wang's team processed Pd(OAc)_2_ and COF‐LZU1, which were used as catalysts for the Suzuki–Miyaura‐coupling reaction. They used COF Pd/COF‐LZU1 materials to construct Pd(OAc)_2_ as the Suzuki catalyst for the Miyaura coupling reaction, which revealed its high efficiency, good stability and recyclability.^[^ ^280^ ^]^ Similarly, two covalent argon skeletons constructed from meso‐tetra‐tra (4‐hydrazidocarbonylphenyllphenyl) and phenyl acid or folate were prepared, and the catalytic performance, showing the selective oxidation of olefins, is embodied by the modification method of manganese (III) ions in COFs.^[^ ^281^ ^]^ In particular, to further prove that these COFs are active as catalysts, Wang's team constructed a combination of 4,4′‐(1H‐benzo[d]‐imidazole‐4,7‐diyl) and various COFs.^[^ ^282^ ^]^ Additionally, Cui's team developed hand‐like 2D COFs by embedding the hand‐in‐hand group into COFs as an efficient multiphase catalyst.^[^ ^283^ ^]^

#### 5.5.2 Gas Storage and Separation

##### COFs

**Figure 39:**
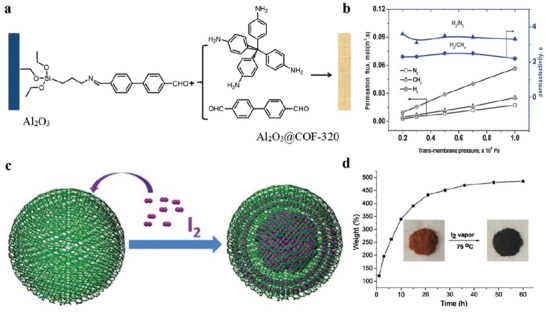
a) Schematic of gas permeability of COF‐320 membranes and b) supported COF‐320 membranes. Reproduced with permission.^[^ ^111^ ^]^ Copyright  2015, Royal Society of Chemistry. c) Display of COF captured by iodine. d) Display of iodine absorption at 75 °C cover time (illustration: COF photos before and after exposure to iodine vapor). Reproduced with permission.^[^ ^288^ ^]^ Copyright 2017, Royal Society of Chemistry.

In terms of application, COFs have broad prospects, especially for gas storage and separation.^[^ ^112^, ^284^, ^285^, ^286^ ^]^ At 298 K and 80 bar, the storage capacities of COF‐320 and methane are 5 wt% and 25 wt% (203 cm^3^ cm^−1^), respectively. The hydrogen absorption values of COF‐102 and COF‐103 are 72.4 and 70.5 mg g^−1^, respectively. Recently, porous *α*‐Al_2_O_3_ ceramics have been prepared by forming a covalent bond between COF‐320, porous *α*‐Al_2_O_3_ carrier and 4,4′‐biphenyldialdehyde formaldehyde. **Figure** **39**a shows the schematic of the structure of the new COF films. The results show that the COF‐320 membrane has H_2_ selectivity, with a pass rate of 5.67 × 10^7^ mol (m^2^ s Pa)^−1^ (Figure 39b), which further proves that the COF membranes can be used in membrane separation.^[^ ^111^ ^]^ The Gascon team prepared COF matrix membranes, which are highly selective to the molar mixture of CO_2_ and CH_4_, and specifically pointed out that these are hybrid matrix membranes comprising COFs connected by matrix amine and nitrogen bonds.^[^ ^287^ ^]^ Zhao's team developed a heterosporous COF (Figure 39c,d) by combining 4,4′‐bis(‐is(4′ fo′my’’henyl) amino), [1′1′:4″,1″‐terphenyl] ‐2′,5′‐dicarbaldehyde and 1,4‐diaminobenzene.^[^ ^288^ ^]^

##### HOFs

**Figure 40:**
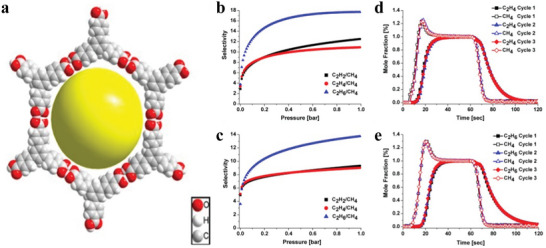
a) 2D hexagonal sheet chip with single‐mode 3‐c HCB topology. The yellow sphere in the middle is a 1D channel with a 25 nm diameter. Separation selectivity of binary gas mixtures at different temperatures: b) 273 and c) 295 K. d) C_2_H_4_‐to‐CH_4_ mole ratio of 1:1 (20 mL min^−1^). e) C_2_H_6_‐to‐CH_4_ mixed mole ratio of 1:1 (20 mL min^−1^). Reproduced with permission.^[^ ^134^ ^]^ Copyright 2013, Royal Society of Chemistry.

HOFs can be used for hydrocarbon separation. Low boiling point gases by low‐temperature distillation consume huge energy.^[^ ^289^ ^]^ Therefore, adsorption separation is considered a potential energy‐saving alternative technology. The high porosity of 2D HOFs significantly enhances their gas absorption rate. Zentner et al. reported that after multiple interpenetrations, some HOFs exhibit high‐strength porosity.^[^ ^124^, ^290^ ^]^ Baek et al. have proved that these HOFs can be used to separate light hydrocarbons (**Figure** **40**).^[^ ^134^ ^]^ At temperatures of 273 and 295 K, HOF‐BTB has a higher adsorption capacity for C_2_ hydrocarbons. Also, the adsorption capacities of C_2_H_4_ and C_2_H_6_ are the best among all currently reported HOFs, whereas the adsorption capacity of C_2_H_2_ is the second‐highest of all HOFs. The breakthrough experiment showed that under the dynamic mixed flow conditions, HOF‐BTB could selectively separate C_2_H_4_ and C_2_H_6_ from CH_4_.

#### 5.5.3 Molecular Adsorption Applications

##### COFs

**Figure 41:**
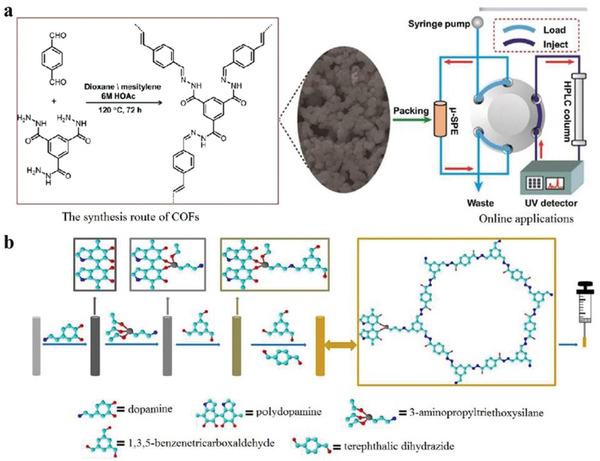
a) Micro solid‐phase extraction system display. Reproduced with permission.^[^ ^293^ ^]^ Copyright 2015, Elsevier B.V. b) Demonstration of SPME fiber synthesis. Reproduced with permission.^[^ ^294^ ^]^ Copyright 2016, Elsevier B.V.

COFs have outstanding characteristics in the adsorption of small molecules, which is particularly important in separation and enrichment. In 2015, Li et al. showed that the new benzodiazepine could be used as a solid phase extractor, which has outstanding performance in the matrix for uranium separation and enrichment.^[^ ^121^ ^]^ The selection of COF‐based solid‐phase extraction agents for uranium is more rigorous. COF‐based materials will be more widely used in functional solid‐phase matrices in the future.^[^ ^291^ ^]^ Similarly, a new 2D ultra‐microporous phosphate‐based COF has great application potential in the selective adsorption of uranium.^[^ ^292^ ^]^ **Figure** **41**a shows a COF‐based micro‐solid phase extraction. This material was designed by Zhang's team to enrich and analyze trace amounts of Sudan dye in paprika and sausage samples.^[^ ^293^ ^]^ COF is a promising medium widely used for sampling, enrichment and separation of target samples.^[^ ^294^ ^]^ Additionally, attractive COF adsorbents made via pre‐treatment technology have several applications (Figure 41b) owing to their ideal enhancement factors and lower detection limits.^[^ ^294^, ^295^ ^]^ Similarly, spherical COFs, which play a prominent role in separating various important industrial drugs in high‐resolution chromatography, were reported by Yan et al.^[^ ^296^ ^]^ which further proves the application of COF materials in the adsorption of small molecules.

##### HOFs

**Figure 42:**
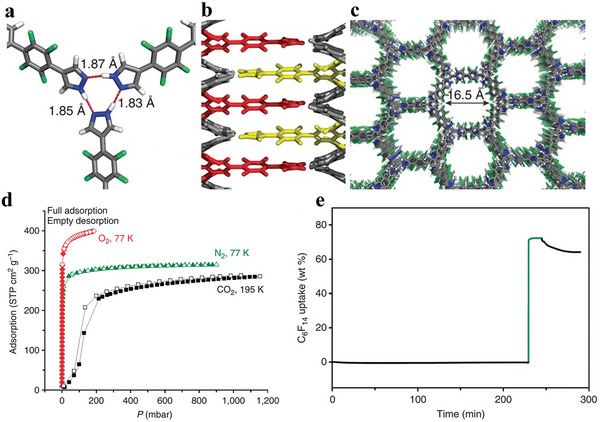
a)Three pyrazoles are brought together in each layer to form triple hydrogen bonds. b) Electron‐rich pyrazole accumulates with the electron‐poor tetrafluorobenzene ring. Consequently, a hexagonal network is obtained, and an infinitely long fluorine‐lined channel runs through the whole structure along the *C*‐axis c) of the crystal. d) Gas adsorption in compound 1 crystals. Crystals of compound 1 absorb N_2_, O_2_ and CO_2_, but not absorb H_2_O vapor, even at 90% relative humidity. e) Time function. Reproduced with permission.^[^ ^130^ ^]^ Copyright 2014, Nature Publishing Group.

HOFs can be used for other volatile adsorbates. Miljanic et al reported the stable adsorption of fluorocarbon (FC) and chlorofluorocarbon (CFC) using an HOF material, which comprises a fluorine‐containing tripyrazole ligand (C_33_H_12_F_12_N_6_, **Figure** **42**).^[^ ^130^ ^]^ In this HOF, the 2D HCB lattice of the hnb topology is given, which is combined by strong *π*–*π* interactions, and the rate of solvent penetration into the hole is 56%. According to the N_2_ adsorption isotherm at 77 K, the BET‐specific surface area of the HOF is 1159 m^2^ g^−1^. This 2D HOF can absorb a negligible amount of water vapor owing to its hydrophobicity. Besides, it can capture a large amount of FCs and CFCs. In particular, at room temperature, a large amount of perfluorohexane (74 wt%) was adsorbed, which revealed its excellent reversibility and fast adsorption kinetics. In another study of theirs,^[^ ^297^ ^]^ by increasing the length of the connector, they successfully obtained several equal structures of HOFs expanded from the prototype, thereby showing their adjustable porosity.

**Figure 43:**
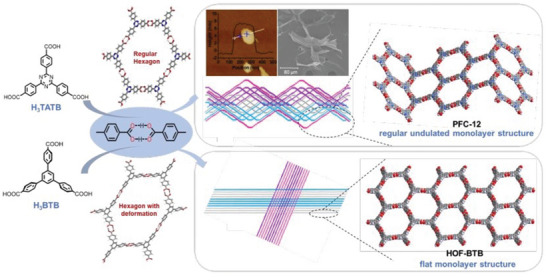
Chemical structure constructed by H3TATB (upper picture) and H3BTB (lower picture), obtained hexagonal HCB pattern and representation of the obtained single‐layer and multichain network. AFM and SEM images of solutes in the supernatant of PFC‐12 crystallization process (fifth and third days, respectively). Reproduced with permission.^[^ ^41^ ^]^ Copyright 2020, American Chemical Society.

2D HOFs can also be used for carbon dioxide capture. The planar molecule H_3_TATB and the nonplanar molecule H_3_BTB with the same hydrogen‐bonding interaction group, but slightly different molecular backbone geometric structures were selected for self‐assembly studies. Liu et al. successfully synthesized three HOF materials: PFC‐11, PFC‐12 and PFC‐13, using H_3_TATB under different solvent conditions (**Figure** **43**).^[^ ^178^ ^]^ In these three structures, adjacent H_3_TATB molecules are connected through double hydrogen bonds, forming regular hexagonal HCB grids. The grid is interwoven and intertwined during formation and expansion.^[^ ^276^, ^298^ ^]^ In contrast, H_3_BTB, which is very similar to the H_3_TATB molecule reported in the literature but non‐planar, also forms a hexagonal HCB grid through self‐assembly. However, the final extension is a flat‐layer oblique interpenetrating structure (HOF‐BTB) without volatility. Their study captured the subversive structural changes caused by the slight twist of CH_4_/H_2_ and conducted in‐depth research from a structural perspective,^[^ ^285^ ^]^ and found that the folds (or undulations) and interspersion in the 2D‐layered structure can be improved. The molecular‐packing density reduces the free energy, thereby achieving structural stability.^[^ ^112^ ^]^ Although PFC‐11, PFC‐12 and PFC‐13 are highly interspersed, the three materials still retain space to accommodate guest molecules, whereas the unique interwoven structure also gives the material some flexibility. PFC‐11 and PFC‐12 show unique breathing effects during nitrogen adsorption. Additionally, PFC‐11 exhibits excellent adsorption capacity for CO_2_. Moreover, because of the size effect of the pores and the structural characteristics of the pore walls, PFC‐11 and PFC‐12 also show good adsorption performance characteristics for organic pollutants benzene, toluene and cyclohexane vapor of comparable pore size. Besides, they have good selective adsorption for benzene vapor with the most matching molecular size and conjugation effect.

##### HCPs

CO_2_ emissions can be transformed by the principle of photocatalysis, but also CO_2_ in the atmosphere can be captured and stored, which also importantly addresses global warming.^[^ ^299^ ^]^ The material of choice often used to improve CO_2_ capture capacity are porous materials. As a micro‐porous polymer, because of its large specific surface area and good stability, 2D HCPs have become one of the choices for CO_2_ storage and adsorption.^[^ ^300^ ^]^ Penchah et al. explored the capture and adsorption capacity of benzene‐based HCP for CO_2_.^[^ ^301^ ^]^ The optimal synthesis time, temperature and pressure were maintained by establishing a thermodynamic model, and the final CO_2_ adsorption capacity value was 262 mg g^−1^. Next, chemical adsorption also has broad prospects in treating water pollution. With the continuous development of industry and society, water pollution has become a very serious problem. Finding an environmentally friendly material to solve the sewage issue is an effective solution.^[^ ^302^ ^]^ Presently, it is recognized that the adsorption of pollutants on substrates can effectively purify the water source. Luna et al. combined HCP particles and composite chitosan (CS) matrix to prepare a hydrogel, effectively removing anionic and cationic dyes from water.^[^ ^303^ ^]^ Also, the composite hydrogel can be reused, and the adsorption capacity will not change. Finally, hydrogen can also be stored by the chemical adsorption method. Since there is no safe and efficient storage method for H_2_, the widespread use of hydrogen is limited.^[^ ^304^ ^]^ Earlier studies reported the physical adsorption process to store hydrogen in porous materials, which has major drawbacks. Therefore, scientists have studied chemically stable 2D organic polymers as hydrogen storage materials. Lee et al. studied a new H_2_ adsorbent based on HCP to enhance the gas transport capacity.^[^ ^305^ ^]^

#### 5.5.4 Biochemistry

**Figure 44:**
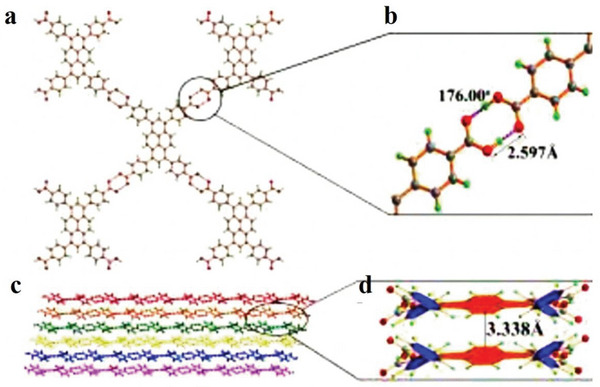
a) Structure of adjacent members. b) Length and angle of hydrogen bonds. c) Stacking of 2D layers. d) *π*–*π* interaction. Reproduced with permission.^[^ ^67^ ^]^ Copyright 2018, Wiley‐VCH.

HOFs: Because HOFs are non‐metal porous media, their excellent biocompatibility and high porosity make them ideal for drug delivery and biomedical applications. Liu et al. designed 2D HOFs with large specific surface area, excellent chemical and thermal stability, and easy regeneration by using pyrene tetracarboxylic acid (H4TBAPy) as the monomer, through multiple hydrogen bonds, strong *π*–*π* interactions and multiple specific surface areas. (**Figure** **44**).^[^ ^67^ ^]^ The BET‐specific surface area of the material was as high as 2122 m^2^ g^−1^, and still maintained its skeletal integrity after soaking in concentrated HCl for 117 days. The self‐recovery property of 2D HOFs under acidic conditions was first discovered. Also, because of the characteristics of metal‐free, low biotoxicity and singlet oxygen generation, doxorubicin, an anticancer drug, was novelly introduced into the pore canal of the 2D HOFs, which successfully realized the chemophotodynamic synergistic treatment of cancer in situ cell experiment. This work provides new ideas for designing and synthesizing stable 2D HOFs, and provides a new way for further developing 2D HOFs and applying them in drug transport and cancer joint treatment.

## 6 Prospects and Challenges of 2D Organic Materials

This study summarizes the latest achievements in the preparation, performance and application of 2D organic materials. However, there are still some challenges in individual technologies that require continuous research to overcome. The prospects and challenges of these five materials are discussed below as follows.

### 6.1 MOFs

In the synthesis of 2D MOFs nanocrystals, two methods are mainly discussed here: top‐down and bottom‐up approaches. These two techniques have their specific characteristics but also have their shortcomings. Presently, the top‐down approach still needs further exploration as the yield of products obtained by this method is low and cannot meet practical applications. Some researchers have tried combining two different approaches to overcome the drawbacks associated with these methods. 2D MOFs synthesized by the top‐down approach have poor stability. The main solution is to add several stabilizers to reduce the lofty interface energy of the MOFs. For the bottom‐up approach, the nanosheets integrated by the interface compound method have low crystallinity, but it has a good application in preparing large‐sized nanosheets. The advantage of the surfactant‐assisted synthesis method is that non‐layered MOFs nanosheets can be synthesized. Because of the stabilizing effect of surfactant, the prepared 2D MOF nanosheets are stable and uniform. However, the active center of the nanosheet prepared by the surfactant‐assisted method is blocked, and the synthesized nanosheet cannot be well applied in catalysis.

2D MOFs can absorb photoelectrons in a certain range and generate photocurrent; thus, they can be used for photocatalysis and optical sensing. The excellent conductivity of 2D MOFs can realize stable charging and discharging process, and can be applied to electrochemical sensors. The porous feature of 2D MOFs can increase the contact area with reactants and improve the catalytic capacity. The application of 2D MOFs in electronic devices is of great interest to researchers; however, there are few successful examples, mainly because the synthesized 2D MOFs are of low quality. Therefore, producing high‐quality materials or developing new preparation methods will become an important challenge in the future. It is also an important aspect of broadening the application of 2D MOF.

### 6.2 COFs

Currently, 2D COFs are emerging porous materials. 2D COFs have obvious characteristics, such as fixed porosity, relatively low density, wide designability and functionality, large surface area, good channel structure, clear pore size and relatively high stability. Owing to these outstanding characteristics, these materials are widely used in photovoltaics, gas storage, catalysis, gas separation, and small molecule adsorption. Based on the above characteristics, there exists a strong research interest.

However, the application of COFs is still limited, mainly due to their insoluble powder and difficult processing characteristics. Their industrial applications are still hindered because of the relatively weak coordination and hydrogen bond interactions associated with relatively low chemical and photophysical stability. Although the outstanding features of 2D COFs in the above reports are very interesting, their processability and the limitations to their industrial applications are the main challenges currently faced by researchers.

Energy and environmental issues are key problems for ecosystem, which can be encountered by using COF due to their unique structural and easy approach to engineering organic materials to provoke energy and environmental problems. In design and synthesis prospective COF, s has several stages. As many COFs have been manufactured in the shape of single crystals, most of the COFs are fabricated as polycrystalline materials. Although the defects formation and their mechanisms are still not well clear, Recently, we know very less about the defects and their 3D distributions. This leads to the preparation of high‐quality COFs crystallites. There is a diversity in COFs synthesis, due to crystallinity, monomer scope and reaction conditions make stable crystalline porous materials is still a significant and challenging venture. Studies on these primary issues stand a great chance to disclose the COFs nature both in structure and property. For various applications, there is tendency to study the properties and functions of COFs. However, due to the micro irregularities and boundaries effects, their functions and properties still needs further research. Also for scale‐up applications, the ordered structure is not sufficient. More importantly, unique property and function are essential to demonstrate that only COFs work for the purpose. In this regard, we are still far from meeting expectations of large‐scale implementations. For energy conversion, COFs have huge advantages in designing *π*‐architectures, such as the COFs’ polymerization making long‐range‐ordered structures to design energy conversion catalysts. Recently we have the challenges that how to full use of the structural topographies to discover the framework so that each procedure is interlocked faultlessly. Concerning the electrocatalytic reduction of carbon dioxide, COFs have shown a great potential of combining conductivity with catalytic activity to promote the transformation. The energy efficiency of the electrocatalytic reduction of carbon dioxide is unknown and needs to be evaluated. This point becomes a key factor in applications. Converting carbon dioxide into value‐added chemicals such as methanol and ethylene deserves further investigations. For applications, a large‐area electrode is necessary; preparing meter‐scale COF electrodes without macroscopic and microscopic defects remains a challenging issue.

### 6.3 HOFs

2D HOFs still face several challenges. Like MOFs and COFs, there is a dire need to accurately control the pore diameter and chemical properties of 2D HOFs. Therefore, to overcome the polymorphism of 2D HOF, first, improving the phase purity is necessary. In addition, it also requires directional synthesis predictably. There are still some challenges in constructing 2D HOFs with excellent performance characteristics, which combine the advantages of ultra‐high surface area and high frame stiffness, and may require new strategies. Owing to the lack of functional sites, especially strong Lewis base–acid sites, the application of 2D HOFs is substantially restricted, and it should be considered when synthesizing and designing future 2D HOFs. The mixed organic ligand crystals are single 2D HOFs and maybe the same or different. Although it is very challenging in this respect, it is of great significance for improving the diversity of the 2D HOF structures. In the future, it will cause significant changes. The new dimensions and fields of 2D HOF chemistry research will enrich and supplement 2D HOF chemistry, thereby providing different functions and applications.

### 6.4 Perovskite

Although many 2D organic perovskites have been studied for decades, the study of the material thickness (from a single layer to tens of nanometers of perovskite crystals) remains a challenge. So far, most laboratories have relied on spin‐coating and mechanical lift‐off methods to prepare thin films for solar cells and LED, which is not very accurate for controlling film thickness. Excitingly, scientists have reported and proved that atomic layer deposition and organic molecular beam epitaxy may be useful for the large‐scale growth of thin 2D organic perovskites. However, further research is needed to bridge the laboratory research and application gap. Owing to the excellent photoelectric properties of 2D organic perovskites, they have been reported in various fields, such as solar cells, LED, synaptic devices, memristors and field‐effect transistors. By introducing a convenient method for large‐scale material synthesis, novel characteristics of 2D organic materials can be further explored, determining the most promising application of 2D organic perovskites in industrial electronic equipment.

### 6.5 Molecular Crystal

Presently, the preparation methods of 2D molecular crystals have been gradually improved. However, the further expansion of this area and the stability and uniformity of the prepared 2D molecular crystal film still have certain difficulties. In the future, it is more necessary to explore the latest technology for synthesizing molecular crystals with better performance. Preparing organic superlattice and quantum well‐structured 2D molecular crystals by adjusting the material structure and optimizing the functional interface is still a big challenge. On this basis, it is necessary to develop more new applications of molecular crystals to promote the development of organic electronic devices. The development potential of 2D molecular crystals in flexible devices is enormous. But so far, there is a lack of reports on the use of 2D molecular crystals in flexible devices. Thus, more research effort is needed to study this application. Finally, the ultimate goal is to use the experimental results in large quantities and at a low cost in industrial production.

## 7 Conclusion

Currently, 2D organic materials are under development, particularly in materials science and nanotechnology. This review systematically introduces the latest progress in the preparation methods, material properties and applications of 2D organic materials. It also addresses the application prospects and challenges of various materials. Owing to their unique optical, electrical, optoelectronic and magnetic properties, 2D organic materials have extensive application prospects in chemistry, biology, electronic devices, micro–nano optoelectronics and sensors. Additionally, the integration of silicon‐based semiconductor technology can considerably promote the development of chip technology, and provide guidance as well as assistance for developing 2D organic materials. As the family of 2D organic materials expands, emerging materials are constantly being discovered, thereby exhibiting their unique properties and laying the foundation for wider research activities and applications. Therefore, with the development of science and technology, 2D organic materials are expected to lead the industrial revolution based on material innovation and promote revolutionary change in information technology.

## Conflict of Interest

The authors declare no conflict of interest.
